# Informing decision-making for universal access to quality tuberculosis diagnosis in India: an economic-epidemiological model

**DOI:** 10.1186/s12916-019-1384-8

**Published:** 2019-08-06

**Authors:** Hojoon Sohn, Parastu Kasaie, Emily Kendall, Gabriela B. Gomez, Anna Vassall, Madhukar Pai, David Dowdy

**Affiliations:** 1 Department of Epidemiology, Johns Hopkins Bloomberg School of Public Health, 615 N. Wolfe St., E6529, Baltimore, MD 21205 USA; 2 Division of Infectious Disease, Johns Hopkins University School of Medicine, Baltimore, MD 21205 USA; 3 Department of Global Health and Development, London School of Hygiene and Tropical Medicine, London, WC1E 7HT UK; 4 Department of Epidemiology & Biostatistics & McGill International TB Centre, McGill University, Montreal, QC H3A 1A2 Canada; 5 Manipal McGill Centre for Infectious Diseases, Manipal Academy of Higher Education, Manipal, India

**Keywords:** Tuberculosis, Diagnostic techniques and procedures, Cost-benefit analysis, Systems analysis

## Abstract

**Background:**

India and many other high-burden countries have committed to providing universal access to high-quality diagnosis and drug susceptibility testing (DST) for tuberculosis (TB), but the most cost-effective approach to achieve this goal remains uncertain. Centralized testing at district-level hub facilities with a supporting sample transport network can generate economies of scale, but decentralization to the peripheral level may provide faster diagnosis and reduce losses to follow-up (LTFU).

**Methods:**

We generated functions to evaluate the costs of centralized and decentralized molecular testing for tuberculosis with Xpert MTB/RIF (Xpert), a WHO-endorsed test which can be performed at centralized and decentralized levels. We merged the cost estimates with an agent-based simulation of TB transmission in a hypothetical representative region in India to assess the impact and cost-effectiveness of each strategy.

**Results:**

Compared against centralized Xpert testing, decentralization was most favorable when testing volume at decentralized facilities and pre-treatment LTFU were high, and specimen transport network was exclusively established for TB. Assuming equal quality of centralized and decentralized testing, decentralization was cost-saving, saving a median $338,000 (interquartile simulation range [IQR] − $222,000; $889,000) per 20 million people over 10 years, in the most cost-favorable scenario. In the most cost-unfavorable scenario, decentralized testing would cost a median $3161 [IQR $2412; $4731] per disability-adjusted life year averted relative to centralized testing.

**Conclusions:**

Decentralization of Xpert testing is likely to be cost-saving or cost-effective in most settings to which these simulation results might generalize. More decentralized testing is more cost-effective in settings with moderate-to-high peripheral testing volumes, high existing clinical LTFU, inability to share specimen transport costs with other disease entities, and ability to ensure high-quality peripheral Xpert testing. Decision-makers should assess these factors when deciding whether to decentralize molecular testing for tuberculosis.

**Electronic supplementary material:**

The online version of this article (10.1186/s12916-019-1384-8) contains supplementary material, which is available to authorized users.

## Introduction

Tuberculosis (TB) is one of the world’s most important infectious causes of morbidity and mortality, with an estimated 25% of the world’s population infected [1] and 1.6 million deaths reported in 2017 [2]. Global TB incidence is declining slowly (2% per year), far short of the progress needed to achieve global targets [2]. With only 64% of an estimated 10 million new cases reported in 2017 [2], strategies to optimize existing technologies and interventions to address gaps in TB case detection and linkage to care are desperately needed.

Xpert MTB/RIF (Xpert), a cartridge-based nucleic acid amplification test (CBNAAT) for TB with substantially improved sensitivity relative to sputum smear microscopy (SSM) [3], has been recommended by the World Health Organization (WHO) [4] and scaled up in many settings worldwide. Many studies have suggested that Xpert is both feasible and cost-effective [5–7] in idealized settings. Nevertheless, in India—as in many resource-limited settings—the use of Xpert has largely been limited to the public sector (at district level or higher [8]) and often restricted to high-priority populations including HIV-positive individuals, children, and people at high risk for multidrug-resistant (MDR) TB [9]. As a result, in more peripheral settings where the majority of public-sector patients present for care (e.g., primary care clinics), TB diagnosis continues to rely on SSM and non-microbiological tests.

Since the initial development of the GeneXpert system, much progress has been made in the development of smaller point-of-care (POC) instruments. In 2018, the battery-operated, single-module GeneXpert Edge system was introduced, and the point-of-care GeneXpert Omni system is expected to be released in 2020. As such, decentralization of Xpert at peripheral facilities using these POC platforms may help address the problem of pre-treatment loss to follow-up (LTFU), which is estimated to occur among 13% of individuals with confirmed TB in Asia [10]. An alternative to decentralization is to centralize Xpert testing at district-level or reference facilities and establish a “hub-and-spoke” transport network for clinical specimens (e.g., sputum) [11]. This approach could enable cost sharing across disease entities, consolidate maintenance of high-level infrastructure in more centralized facilities, and ensure higher testing volume to achieve economies of scale. South Africa, for example, has successfully used this centralized model via their National Health Laboratory Service (NHLS) [12].

Given the resource constraints and the complexity and heterogeneity of most healthcare systems, it is important to estimate the impact and cost-effectiveness of more centralized Xpert testing versus decentralizing Xpert testing capacity to more peripheral settings. This is also a question of generalizable importance, as many other tests on the WHO Essential Diagnostics List (e.g., HIV viral load testing, glycated hemoglobin measurement for diabetes) could be performed either centrally or in point-of-care fashion [13]. To help illustrate the primary considerations in evaluating the cost and cost-effectiveness of more decentralized versus more centralized Xpert testing, we constructed a suite of economic and epidemiological models in a hypothetical setting representative of the Indian public sector.

## Methods

### Experimental framework

Our primary comparison was between more centralized Xpert testing for TB at district/city-level hubs with supporting sample transport networks (“centralized” scenario) and decentralization of Xpert testing to more peripheral settings in clinics where microscopy currently is performed (“decentralized” scenario) within India’s current public sector system, namely the Revised National Tuberculosis Control Program (RNTCP) [14]. In the decentralized scenario, we assumed that Xpert is made available (for example, using battery-operated Edge instruments, or Omni, in the near future) at the Microscopy Center (MC), a primary health center serving approximately 100,000 people with a capacity to perform SSM. In this scenario, we assumed a sufficient number of instruments installed to ensure the same-day diagnosis of > 90% of all patients with presumptive TB.

**Fig. 1:**
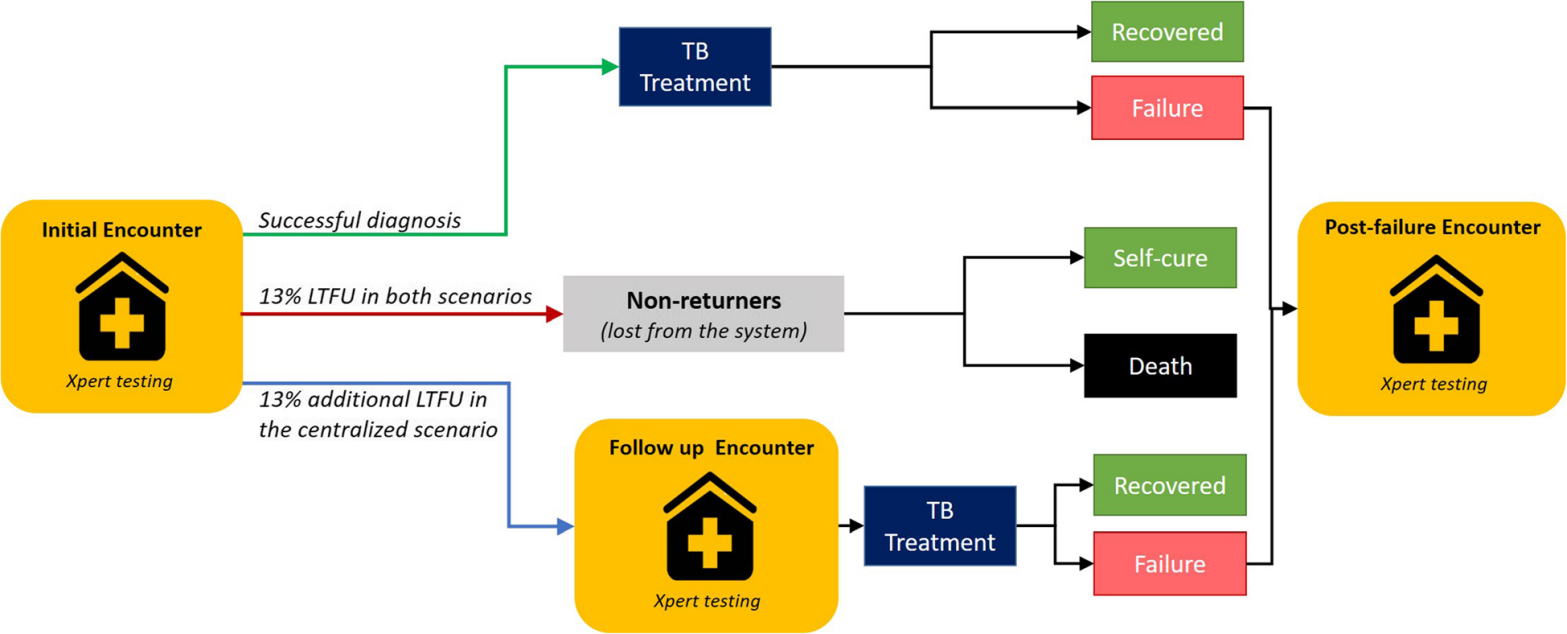
Schematic representation of the tuberculosis (TB) diagnostic process in the public sector. Upon accessing the public sector for TB diagnosis, individuals are assumed to undergo an initial evaluation that includes Xpert MTB/RIF (or sputum smear microscopy prior to Xpert implementation). A proportion of those encounters will result in successful diagnosis followed by successful treatment (green arrow) or pre-treatment loss to follow-up (LTFU) estimated at 13% for both centralized and decentralized scenarios (red arrow). These individuals may undergo other diagnostic encounters, but those encounters do not result in TB diagnosis, and the cost of those encounters is not considered by the model. In addition to these individuals, another proportion of individuals (also estimated at 13% in the base case) are expected to experience pre-treatment LTFU due to diagnostic delays in the centralized scenario (blue arrow). These individuals can return for a future visit in which Xpert is again performed and TB treatment is successfully initiated. The assumption that all incremental LTFU is followed by a second encounter is conservative and may underestimate the impact of Xpert in the decentralized scenario. Finally, individuals receiving TB treatment who fail to recover will undergo a separate encounter after treatment completion in which Xpert is performed and used to guide second-line therapy

In the centralized scenario, we assumed that Xpert is available only at the District TB Center (DTC), a central referral facility currently equipped with four-module GeneXpert IV instruments that cannot readily be implemented at the point of care. There are more than 13,000 MCs in India, with a median of 13 MCs (interquartile range (IQR) = 9–23) associated per DTC. We assumed that sputum specimens are transported from individual MCs to the DTC using a specimen referral network. In both decentralized and centralized scenarios, we consider only patients who present to peripheral centers (i.e., MCs) for testing, as decentralization will not affect outcomes of patients presenting directly to district-level centers (i.e., DTCs) for diagnosis. In both scenarios, we assumed that 13% of individuals presenting to care would experience LTFU prior to treatment initiation and experience no benefit from TB testing (Fig. 1, red arrow). These individuals may undergo other diagnostic encounters, but the cost and outcome of those encounters are assumed not to differ between scenarios (and thus not explicitly considered) [10]. Since referral tests generally cannot provide same-day results, we assumed that centralized testing would result in additional pre-treatment LTFU for some patients who would receive same-day treatment in the decentralized scenario (Fig. 1, blue arrow); we assume that these patients will return for follow-up visits and obtain an appropriate (though delayed) diagnosis in the future. In both strategies, Xpert was assumed to be used as the primary diagnostic tool for all presumptive TB patients, a key objective of India’s 2025 National Strategic Plan to ensure universal and upfront access to molecular diagnostics for TB.

To evaluate cost-effectiveness, our a priori assumption was that decentralized testing would be more expensive (as it would require more equipment modules), but also more effective (as it would reduce pre-treatment LTFU). To evaluate this trade-off, we first constructed a range of cost functions to estimate the unit cost of Xpert from the health system perspective, under various scenarios. We then adapted a published agent-based simulation of drug-susceptible (DS) and drug-resistant (DR) TB transmission in India [15] to estimate the incremental epidemiological impact and cost-effectiveness (assessed as the cost per disability-adjusted life year [DALY] averted) of decentralized versus centralized Xpert.

### Cost model

Using a bottom-up micro-costing method incorporating data from our previous work in India [5, 16], we constructed a reference per-test cost table for Xpert (see Additional file 1: Section S1.2). We estimated the price of a peripheral single-module GeneXpert unit as US$2895, with similar per-module maintenance pricing as the GeneXpert IV [17]. We inflated all Xpert-related (equipment and cartridge) unit prices by 20% to account for procurement and installation. We assumed that the amount of time required to perform an Xpert test did not differ with centralized versus decentralized testing, as the procedures are standardized, require minimal hands-on time, do not differ based on the type of GeneXpert system used, and can be operated by existing laboratory staff (e.g., microscopy technicians who currently perform smear testing at MCs). We also assumed the equivalent quality of testing, as decentralized testing is unlikely to be accepted in settings where quality is substantially compromised. We therefore incorporated costs of continuous quality control measures under each strategy.

Anticipating that laboratory workload (number of tests per day) would strongly influence the unit cost of Xpert [5, 18], we simulated a range of daily testing volumes for MCs and DTCs assuming a Poisson distribution of tests per day and 250 annual operational days. Mean per-test cost was assessed for each volume scenario based on annual total cost and testing volume (see Additional file 1: Section S1.3).

**Fig. 2:**
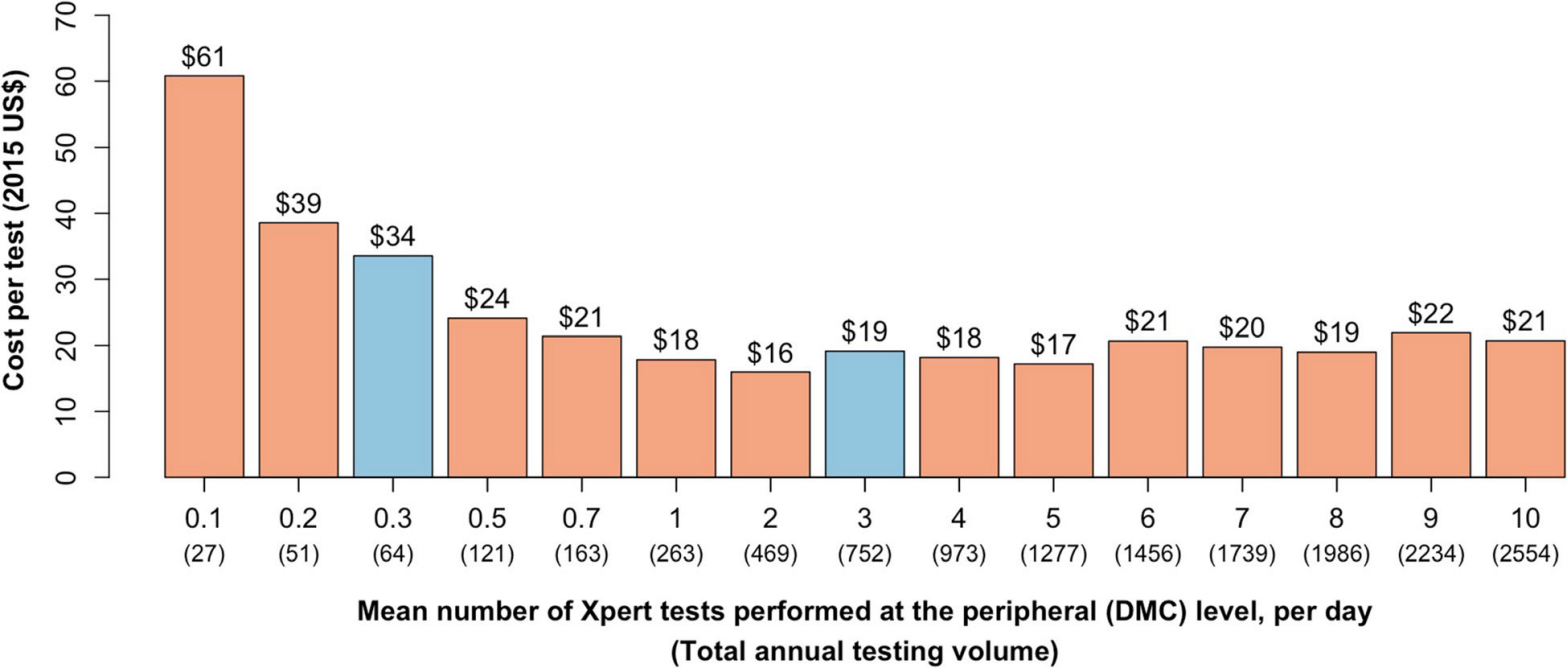
Unit cost of decentralized Xpert testing. Bars show the estimated unit cost (in 2015 US dollars) of decentralized Xpert testing as a function of the mean daily number of tests performed (*x*-axis), with the annual test volume shown in parenthesis under each scenario and the cost per test shown above each bar. Blue bars indicate two representative scenarios chosen for the cost-effectiveness analysis. Costs were derived by assuming a Poisson distribution of daily test volume and number of single-module Xpert devices sufficient to ensure same-day testing for 90% of all patients. Above mean daily volumes of approximately one test per day, the unit cost remains relatively stable (between $15 and $20 per test)—reflecting the fact that, even though a single-module device can perform four tests per day, testing capacity must be maintained at approximately four times the actual volume in order to assure same-day diagnosis at the 90% level. At less than one test per day, excess (wasted) testing capacity grows, resulting in substantially increased unit costs at very low testing volumes

For decentralized testing, we evaluated 15 different scenarios (Fig. 2) with mean daily testing volumes between 0.1 (i.e., 25 samples tested per year) and ten (2500 samples/year). This range covers the expected mean daily workload of 3–6 tests per MC nationwide [14]. For centralized testing, a total of seven mean daily testing volume scenarios between 10 (2475 samples/year) and 70+ (17,411 samples/year) were assessed, including costs associated with sample transport (see Additional file 1: Section S1.4). At the time of analysis, no published evidence on costs of establishing or operating sample transport networks for sputum specimens was available, to our knowledge. Therefore, we estimated mean per-sample transport costs based on the following: (a) distributions of MC-to-DTC ratio and mean distance from MCs to DTC (based on total service area of DTC/number of MC in the area) in India (see Additional file 1: Figure S2), (b) hypothetical estimates of the number of specimens transported, and (c) a range of cost sharing of sample transport (5 to 100%) with other clinical specimens (Additional file 1: Table S4). This cost was calculated separately for each DTC test volume scenario (Additional file 1: Table S6).

Costs for clinical encounters (outpatient visits, clinical evaluation, antibiotic treatment, etc.), TB-specific treatment, and chest X-ray were estimated from sources including the Global Drug Facility, the MSH International Price Tracker, the World Health Organization’s CHOosing Interventions that are Cost-Effective (WHO-CHOICE) database [19], and published literature (Table 1) [22, 23]. Full details of the costing approach are described in Additional file 1: Section S1.

**Table 1:** Selected model parameters for post-calibration sensitivity analysis

Parameters	Base value	Low bound^†^	High bound	Reference
Xpert diagnostic accuracy				
Sensitivity—smear positive	0.98	0.93	1.00	[3]
Sensitivity—smear negative	0.67	0.60	0.85	
Sensitivity—RIF resistance	0.95	0.90	0.97	
Specificity—diagnosis of TB disease	0.99	0.98	1.00	
Specificity—RIF resistance	0.98	0.97	1.00	
Treatment and linkage to care				
Probability of empiric treatment at initial encounter	0.25	0.00	0.50	Assumption
Probability of loss to follow-up after initial encounter	0.13	0.05	0.25	[10]
Monthly rate of returning for follow-up encounter	0.46	0.30	0.70	[20]
Probability of treatment failure for drug-resistant TB	0.17	0.10	0.25	WHO 2016
Probability of referring from private provider to public sector	0.67	0.54	0.80	[21]
Type of costs				
Cost of initial encounter	$12.11	$9.08	$15.14	Recalculated based on [19, 22, 23]
Cost of follow-up encounter	$9.53	$7.15	$11.91	
Cost of encounter to assess failing treatment	$7.39	$5.54	$9.24	
Other visits (informal/private)	$2.32	$2.06	$2.60	
DS-TB treatment, per 6-month course	$345.65	$259.24	$432.06	
DR-TB treatment, per course	$3399.22	$2549.41	$4249.02	

^†^One-way sensitivity analysis was performed by varying each parameter in this table individually between its low and high bounds. For a list of all parameters and references, see Section S2.6 of Additional file 1

### Agent-based simulation model

We developed an agent-based simulation model of TB transmission and treatment in India, incorporating household structure, patients’ care-seeking pathways, and the public-sector TB care cascade (Fig. 1, Additional file 1: Figures S4 and S5). A detailed description of the model is presented in Additional file 1: Section S2 and earlier publications [15, 20, 21, 24]. Briefly, the simulation model was calibrated to 2017 WHO estimates of TB incidence, prevalence, and mortality in India using a stepwise procedure (see Additional file 1: Section S2.5). At the end of the calibration period, we modeled the introduction of Xpert in area(s) where Xpert testing is not available, as a first-line test replacing on-site SSM, under decentralized and centralized testing scenarios as described above. The model was then run for 10 years to project corresponding changes in the incidence and mortality of DS- and DR-TB. The core model simulates a population of one million people, representative of an average-sized district in India that might contain a single DTC [25]. For each Xpert testing scenario (centralized versus decentralized), we generated 2000 independent simulations of TB transmission at the district level. To assess the policy implementations at a city or state level, we then constructed larger simulated populations of 20 million people by combining 20 randomly sampled districts (with replacement) from the original pool of simulations. We repeated this procedure to achieve a sample of 2000 larger-scale simulations with a population of 20 million individuals each. All outcomes were reported as median values with interquartile uncertainty ranges across these 2000 larger-scale simulated populations.

### Cost-effectiveness analysis

**Fig. 3:**
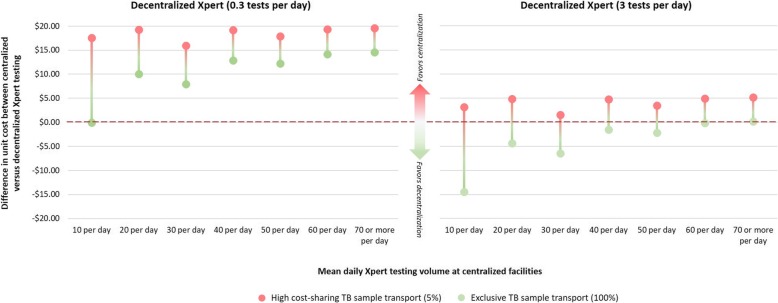
Difference in unit cost of more centralized versus more decentralized Xpert testing for tuberculosis (TB). Shown on the *y*-axis is the range of difference in mean per-test cost of Xpert test (inclusive of equipment, consumables, sample transport, human resources, and overheads) performed in the centralized scenario (district-level testing with sample transport network, assuming a range between 5 (“high cost sharing”) and 100% (“exclusive TB sample transport”) of this network—represented by the size of each bar—is used for sputum specimens/TB testing) and in the decentralized scenario (testing at the microscopy center level). Values above the horizontal line ($0, gray dotted line) denote that decentralized testing is more expensive than centralized testing, whereas values below that line denote that centralized testing is more costly. The dumbbell dot plot on the left represents a setting where a mean of 0.3 tests are performed per day at the peripheral level (i.e., smaller, low-volume clinics), whereas those on the right represent a mean volume of 3 tests per day. The seven boxes in each series represent, from left to right, increasing mean test volume of 10 to 70 or more tests per day at the centralized testing facility. Such variation might be seen, for example, if an increasing number of peripheral clinics referred specimens to the same district center, or if the district center performed testing for other purposes (e.g., inpatients, drug susceptibility testing). In general, increasing testing volume makes maximal use of equipment and sample transport network costs, such that higher test volumes in the periphery favor decentralized testing, whereas higher test volumes at the district level favor centralized testing. Full assessment of all scenarios is available in Additional file 1: Section 1.5 and Figure S3

Given our a priori assumption that decentralized testing would be implemented with equal quality and would reduce pre-treatment LTFU with no adverse consequences (i.e., that decentralized testing would be more effective than centralized testing and thus preferred in settings where decentralized testing was less costly), we focused our cost-effectiveness analyses on our tested scenarios where the unit cost of decentralized testing would be higher than that of decentralized testing. We selected these scenarios based on their conceptual importance: one scenario of very low decentralized test volumes (0.3 tests per day, as might be seen in more sparsely populated districts, or as TB incidence declines) and one of average test volumes (three per day, reflective of mean MC volumes in India) [14]. We compared these decentralized scenarios against centralized testing at high volume (> 70 tests per day) with either low or high levels of shared transport costs (see Fig. 3).

All analyses were performed from the health system perspective and assessed in 2015 US dollars. The primary outcome was calculated as the ratio of incremental costs per disability-adjusted life year (DALY) averted over a 10-year period, with costs and DALYs discounted at 3% annually. We benchmarked cost-effectiveness against a range of cost-effectiveness thresholds (CET) from $500 to $2000 to reflect more realistic local constraints than reflected in traditional per capita Gross Domestic Product (GDP)-based thresholds [26, 27].

### Sensitivity analyses

We performed univariate sensitivity analyses for selected epidemiological and cost-related parameters across the ranges shown in Table 1. For each analysis, we evaluated the relative epidemiological impact (reduction in DS-TB incidence), difference in cost, and incremental cost-effectiveness, comparing decentralized to centralized testing. For these analyses, we assumed a decentralized scenario with moderate volume of three tests per day and a centralized scenario with no cost sharing for sample transport. We conducted other sensitivity analyses in which we separately varied incremental pre-treatment LTFU in the centralized scenario, diagnostic accuracy of Xpert, and other drivers of the unit cost per Xpert test (e.g., estimated useful life).

## Results

The unit cost of decentralized Xpert ranged from $17.20 to $60.81 (Fig. 2), but consistently approximated $20 per test when MCs performed at least one test a day (with small fluctuations reflecting the number of testing modules required). The unit cost of centralized Xpert depended on the degree to which the costs of sample transport could be shared with clinical specimens for non-TB disease entities (e.g., from $14.00 to $18.96 in the highest-volume scenario in Additional file 1: Table S8).

The per-test cost difference between centralized and decentralized testing was driven primarily by three factors: centralized (DTC) testing volume, cross-disease cost sharing of specimen transport under centralized testing (between 5 and 100% of the sample transport network dedicated for TB/sputum specimens), and peripheral (MC) testing volume. At higher decentralized testing volume (mean daily testing volume of 3 or more at MCs, represented by *λ* = 3), differences in costs of between centralized and decentralized testing (Fig. 3) were generally small or favored decentralized testing (as indicated by a negative cost estimate). At lower decentralized testing volumes (MC mean daily testing volume of less than 1 test, for example, *λ* = 0.3), centralized testing was generally cheaper than decentralized testing. In both cases, higher sample transport cost sharing and testing volumes for centralized testing lowered the unit cost of centralized testing. Additional description of the calculation of unit costs and sensitivity analyses around the drivers of these costs are shown in Additional file 1: Section S1.6.

### Epidemiological impact of decentralized and centralized Xpert

**Fig. 4:**
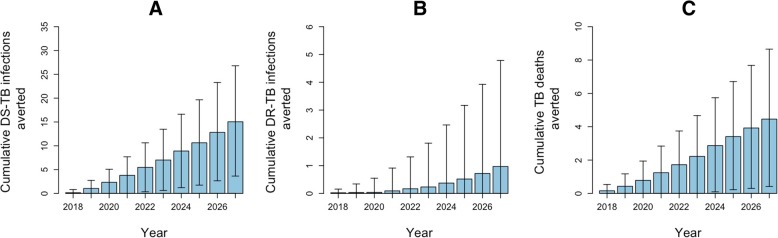
Projected incremental epidemiological impact of decentralized versus centralized Xpert testing for tuberculosis (TB). Panels compare the impact of Xpert implemented via a decentralized strategy compared to centralized testing, in terms of the cumulative number of DS-TB infections averted (**a**), cumulative number of drug-resistant (DR) TB infections averted (**b**), and cumulative number of TB deaths averted (**c**). All values are adjusted to a population size of 100,000. Shaded areas and arrows represent interquartile uncertainty ranges

Assuming that centralized Xpert was used to replace SSM starting in 2018 (with 13% pre-treatment LTFU relative to decentralized testing [10]), our model projected a median TB incidence of 213 [IQR 212–214] cases per 100,000 in 2028 in a hypothetical region representative of India’s public sector where Xpert testing was previously unavailable. Alternatively, if Xpert was implemented in decentralized fashion (with equal quality and no pre-treatment LTFU) in the same region, the resulting DS-TB incidence in 2028 was projected to be 1.0% [IQR 0.5–1.6%] lower than with centralized testing, equivalent to averting a total of 15 [IQR 4–27] new DS-TB cases per 100,000 population over 10 years (Fig. 4a). For DR-TB incidence and TB mortality, the difference between the two strategies was small, with decentralized testing averting an estimated 1 [IQR − 1 to 5] incident DR-TB case and 4 [IQR 0–9] total TB deaths per 100,000 over 10 years (Fig. 4b, c).

### Incremental cost-effectiveness of decentralized versus centralized Xpert

**Fig. 5:**
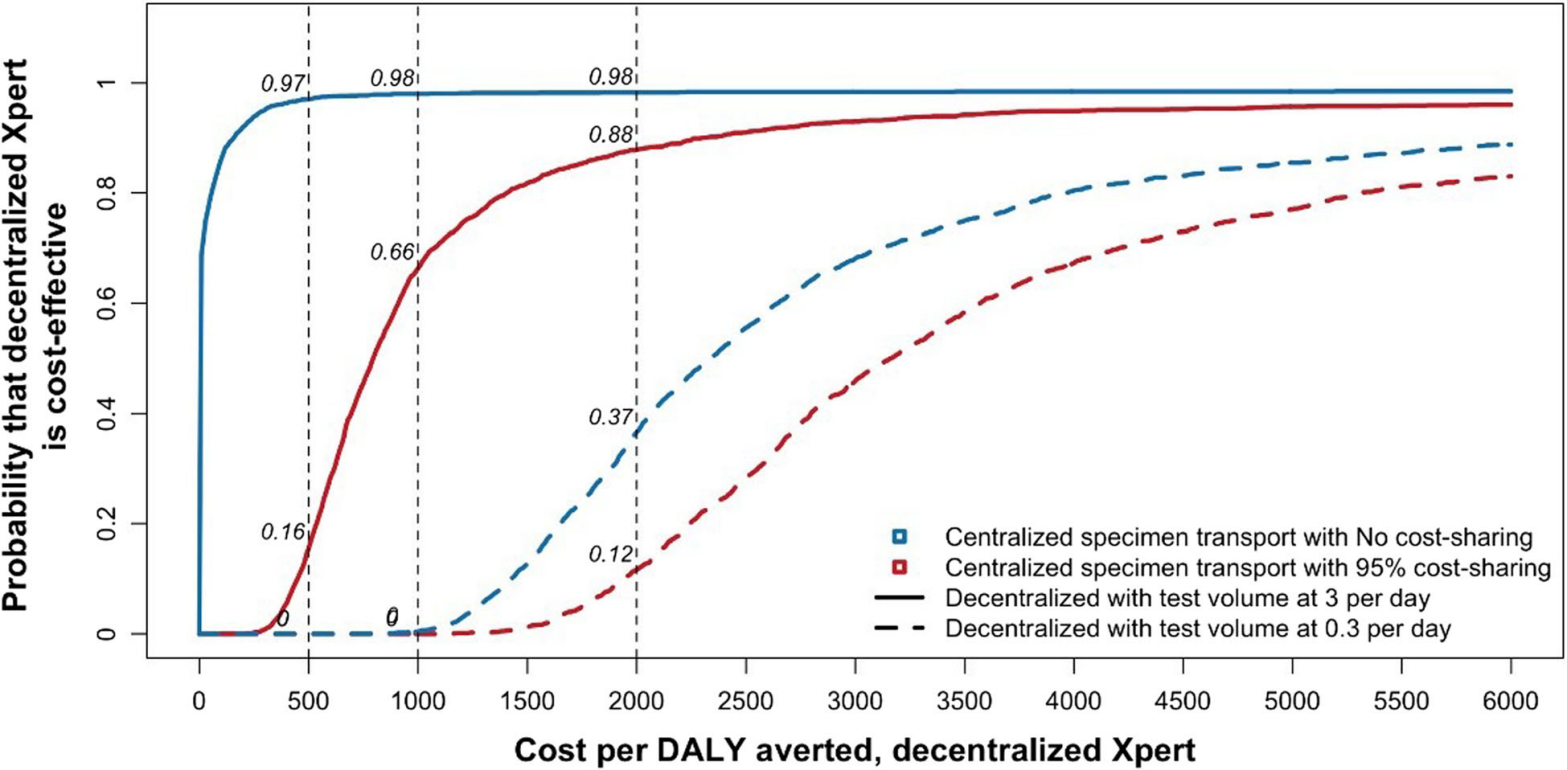
Cost-effectiveness acceptability curves comparing decentralized versus centralized Xpert testing for tuberculosis (TB) in a representative simulated Indian population. The *x*-axis shows the cost per disability-adjusted life year (DALY) averted via decentralized Xpert compared to centralized testing, and the *y*-axis shows the proportion of stochastic simulations falling below each corresponding cost-effectiveness threshold. Dotted vertical lines represent alternative thresholds for evaluating cost-effectiveness. For each threshold, the cost-effectiveness values are computed for the four unit-cost comparisons when centralized specimen transport is utilized at no vs. 95% cost sharing and mean peripheral testing volume ranges from 0.3 to 3 per day in the decentralized scenario

Primary cost-effectiveness results are given in Table 2 and Fig. 5. In the primary analysis, decentralized Xpert averted an estimated 1.25% [IQR 0.88%, 1.54%] of all TB-related DALYs, relative to centralized Xpert. Under the most unfavorable scenario for decentralized testing (low decentralized volume at 0.3 tests per day and 95% cost sharing for specimen transport in the centralized scenario), this gain came at an estimated cost of $3161 [IQR $2412; $4731] per DALY averted. Under the most favorable scenario (high decentralized volume at 3 tests per day and no cost sharing), decentralized testing was cost saving compared to centralized testing, saving a median of $338,000 [IQR − $222,000; $889,000] per 20 million people.

**Table 2:** Incremental cost-effectiveness of decentralized versus centralized Xpert testing

	Centralized Xpert with 95% cost sharing for specimen transport		Centralized Xpert with no cost sharing for specimen transport	Decentralized Xpert with test volume at 3 per day	Decentralized Xpert with test volume at 0.3 per day
Total costs (2015 US$, in million)	$189 [$157–$222]		$196 [$164–$230]	$196 [$164–$230]	$217 [$185–$253]
Total DALYs	740,124 [700,676–782,488]			730,923 [692,001–773,630]	
Comparing centralized vs. decentralized Xpert					
Centralized Xpert	vs.	Decentralized Xpert	Difference in costs (2015 US$, in million)	Difference in DALYs	Cost per DALY averted (2015 US$)
95% cost sharing for specimen transport	vs.	Test volume at 3 per day	$7 [$6.6–$8]	9.26 [6231–12,112]	$795 [$573–$1213]
No cost sharing for specimen transport	vs.	Test volume at 3 per day	− $0.33 [− $0.89 to $0.22]		Dominated* [dominated*–$29]
95% cost sharing for specimen transport	vs.	Test volume at 0.3 per day	$28 [$27–$30]		$3161 [$2412–$4731]
No cost sharing for specimen transport	vs.	Test volume at 0.3 per day	$21 [$20–$22]		$2339 [$1775–$3501]

A total of four cost sets were compared in the cost-effectiveness analysis. All values are expressed as median with interquartile ranges in brackets, cumulative and discounted over a 10-year time horizon

*In the median simulations, decentralized Xpert was less costly and more effective than centralized testing

The probability that decentralized Xpert would be cost-effective depended strongly on decentralized testing volume, degree of cost sharing for centralized specimen transport, and the cost-effectiveness threshold. For example, the probability that decentralized testing would be cost-effective relative to centralized testing ranged from 0.12 to 0.98 at a willingness to pay of $2000 per DALY averted (Fig. 5). At very low peripheral testing volumes (≤ 0.3 tests per day), decentralized Xpert was unlikely to be cost-effective: even at a willingness to pay of $6000 per DALY averted, the probability of cost-effectiveness remained below 0.8.

### Sensitivity analyses

**Fig. 6:**
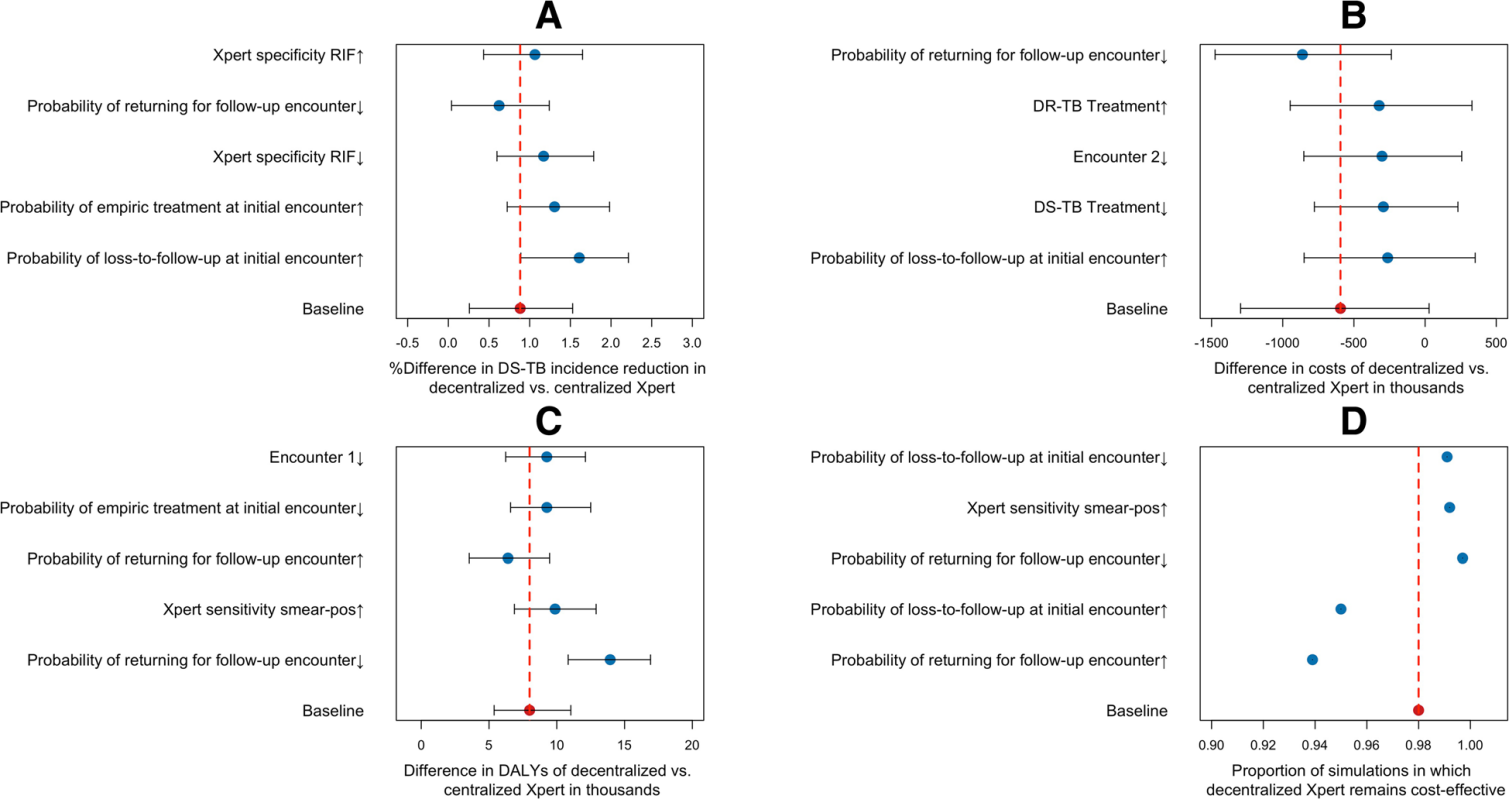
One-way sensitivity analysis to the value of selected model parameters. Panels show the sensitivity of epidemiological outputs (reduction in DS-TB incidence in panel **a**), costing outcomes (difference in costs in panel **b**), and DALYs averted (in panel **c**) comparing decentralized to centralized TB testing, and the proportion of simulations that remain cost-effective at a threshold of $1000 (panel **d**) under one-way variation in the value of selected model parameters. Each parameter value is followed by an up/down arrow, denoting an increase (↑) or decrease (↓) in the input parameter value as listed in Table 1. Each scenario is simulated starting in the year 2018 and is followed to the year 2028 under centralized and decentralized Xpert placement. The bars and arrows represent the median and interquartile ranges across 1000 simulations. The red mark and dashed line represent the baseline scenario with no parameter variation. The results are summarized by showing the five parameters for which variation resulted in the largest variations from the baseline scenario (increasing in impact from top to bottom)

When comparing decentralized against centralized testing, differences in outcome measures—epidemiological impact of Xpert (Fig. 6a), costs (Fig. 6b), and DALYs (Fig. 6c)—were generally robust to one-way variation in parameter values. Incremental cost-effectiveness ratios (Fig. 6d) likewise did not vary substantially in one-way sensitivity analyses, with the uncertainty boundary remaining within ± 10% of the primary estimate. Increasing the amount of pre-treatment LTFU in the centralized scenario reduced the epidemiological impact of Xpert and resulted in higher costs and less favorable cost-effectiveness. For example, when increasing the incremental probability of pre-treatment LTFU with centralized (versus decentralized) testing from 0 to 20%, the proportion of simulations favoring decentralized Xpert (assuming moderate decentralized volume, 95% centralized cost sharing, and a willingness-to-pay threshold of $1000 per DALY averted) rose from 57 to 99%. Complete details of these analyses are given in Additional file 1: Sections S3.1 and S3.2.

Performing SSM as a back-up test in addition to Xpert (thereby allowing same-day diagnosis for all smear-positive patients) improved the epidemiological impact and cost-effectiveness of centralized testing. For example, assuming moderate decentralized testing volume and no cost-sharing for specimen transport, the probability that decentralized testing would be cost-effective relative to centralized testing was estimated as 0.61 ***(compared to 0.98 at baseline). For complete one-way sensitivity analyses results, please see Additional file*** 1***, Section S3.3***.

## Discussion

This economic-epidemiological model of Xpert implementation in India suggests that the economics of decentralizing molecular testing for TB likely depend on three important factors: the testing volume at decentralized facilities and centralized facilities (i.e., how many decentralized facilities are referring), the degree to which costs of specimen transport can be shared with other non-TB disease entities, and the level of pre-treatment LTFU caused by the delays in centralized testing. Provided that decentralized testing can be performed with equal quality as centralized testing, the costs and LTFU incurred by using a hub-and-spoke system are likely to justify the increased costs of decentralized testing in most scenarios, except in settings where testing volumes in the periphery remain very low (mean < 1 test per day).

Our results may appear contrary to existing arguments that point-of-care diagnosis is much more expensive than centralized testing [28, 29]. Our findings reflect, in part, the smaller capital outlay required for smaller GeneXpert Omni and Edge devices: by enabling flexibility in procuring the number of modules based on testing demands at peripheral centers at approximately one fourth the price of a four-module device, these devices enable smaller peripheral centers to perform point-of-care testing without a substantive increase in unit costs. However, in settings where the volume of decentralized testing is very low (high costs associated with peripheral testing infrastructure) and an established low-cost sample referral network exists, optimized hub-and-spoke centralized testing is more likely to be cost-effective. Likewise, it may be financially infeasible to provide peripheral Xpert in all settings (for example, India’s 2025 National Strategic Plan [14] aims to provide Xpert in 8335 MCs). Thus, our results advocate for prioritizing decentralization of rapid TB CBNAAT in regions with higher-than-average MC testing volumes and where sample transport systems do not exist for TB or other diseases while strengthening hub-and-spoke systems (and continuation of smear microscopy) in regions with lower testing volumes in the periphery.

In considering the implementation of molecular diagnostic testing for infectious diseases, it is important to weigh cost-effectiveness alongside considerations of epidemiological impact and feasibility. Our results indicate that the epidemiological impact of decentralized Xpert, while modest, is sufficient to justify its incremental cost in settings characterized by high pre-treatment LTFU, provided that decentralized testing can be feasibly implemented without a loss in quality. However, decentralized testing alone is unlikely to transform TB epidemics in high-burden and heterogeneous settings like India. Therefore, in places where patients are likely to return for follow-up evaluation, parallel investments in strengthening the centralized Xpert system is of equal importance.

Furthermore, with a wide range of novel TB diagnostics (designed for both more peripheral or more centralized placement) [30] approaching market entry, placement strategies for these new technologies will become an important policy debate as countries push to increase the coverage of quality TB diagnosis and care. Our results illustrate that, for technologies optimized for higher level laboratories (e.g., whole-genome sequencing), ability to ensure high testing volumes at the central level and to share health system infrastructure (e.g., specimen transport network) will be important determinants of cost utility. For diagnostics designed for more peripheral placement (e.g., Xpert Edge and Omni, Molbio Truenat MTB), important cost-effectiveness considerations will also include the ability to achieve similar diagnostic performance with simplified logistical infrastructure. As countries plan investments to strengthen the TB care cascade, cost and impact of technologies optimized for different placement strategies must be carefully evaluated against TB control priorities, budget, and current health systems constraints.

As with any modeling exercise, our study has certain limitations. We adopted a number of simplifying assumptions, including homogeneous mixing, representation of India’s TB diagnostic landscape as having only three tiers (informal, formal private, and public), and representation of the diagnostic process as occurring within a discrete number of specific encounters. To the extent that these simplifications depart from the complex reality of TB transmission and diagnosis in the Indian healthcare system, we may over- or underestimate the epidemiological impact and cost-effectiveness of decentralized testing. The most important assumptions in this model are the degree to which decentralized Xpert testing will reduce those who will be permanently lost in the TB care cascade (pre-treatment LTFU, relative to centralized testing) and the degree to which patients will return for follow-up evaluation. Given the complexity of the TB care cascade and patient care-seeking behaviors [31], implementation of decentralized testing alone may not achieve substantive reductions in LTFU [32]. We also did not consider costs of implementing programmatic interventions that may be necessary to translate peripheral testing into reductions in LTFU; where such interventions are required (or where LTFU is already low, even with centralized testing), the cost-effectiveness of decentralized Xpert may be much less favorable than projected here. Nevertheless, we estimated that decentralized testing could be implemented at a lower unit cost than centralized testing in many situations—and to the extent that this can be accomplished (without a loss in quality), decentralized testing is likely to be preferred. Finally, we assumed, for the purpose of comparison, a setting in which Xpert was scaled up as a first-line test for TB among all adults with symptoms; cost-effectiveness of Xpert overall (and of decentralized Xpert relative to centralized Xpert) may be improved by adoption of other algorithms, such as focusing on high-priority populations [33].

## Conclusions

We have used a combined epidemic-economic model to illustrate that decentralized Xpert testing for TB is likely to be a cost-effective strategy in settings characterized by high LTFU, moderate-to-high peripheral testing volumes, and inability to share costs of specimen transport with other disease entities. Our findings reflect the fact that single-module devices enable testing to be performed at lower volumes without considerable increases in per-test costs. Decisions to implement decentralized testing should not be based solely on cost-effectiveness but must also consider budget constraints, feasibility of implementation without a loss in quality, and the degree to which such testing is likely to reduce LTFU in actual practice. Assuming these factors can be addressed, this modeling analysis supports the scale-up of decentralized Xpert testing for TB as a mechanism to achieve universal access to high-quality TB diagnosis in India and other similar settings.

## Additional file

Additional file 1: Detailed methodology for cost analyses, simulation model development, and sensitivity analyses. (DOCX 2.10 mb)

## Data Availability

All data generated or analyzed during this study are included in this published article [and its Additional file].

## Abbreviations

CBNAAT: Cartridge-based nucleic acid amplification test
CET: Cost-effectiveness threshold
DALY: Disability-adjusted life year
DR: Drug resistant
DS: Drug susceptible
DTC: District TB Center
IQR: Interquartile range
LTFU: Loss to follow-up
MC: Microscopy Center
RNTCP: Revised National Tuberculosis Control Program
SSM: Sputum smear microscopy
TB: Tuberculosis
Xpert: Xpert MTB/RIF
