# Supplementary material for: Informing decision-making for universal access to quality tuberculosis diagnosis in India: an economic-epidemiological model
Source: BMC Med. 2019 Aug 6;17:155. doi: 10.1186/s12916-019-1384-8 (PMC6683370; doi:10.1186/s12916-019-1384-8)
Supplement: Supplementary file 1 — Detailed methodology for cost analyses, simulation model development, and sensitivity analyses. (DOCX 2.10 mb) [file 12916_2019_1384_MOESM1_ESM.docx]

Additional File 1

**Supplementary materials to manuscript:**

**Informing decision-making for universal access to quality tuberculosis diagnosis in India: an economic-epidemic model**

Authors: Hojoon Sohn, PhD ^1^ Parastu Kasaie, PhD^1^ Emily Kendall, MD^2^ Gabriela B Gomez, PhD^3^

Anna Vassall, PhD^3^ Madhukar Pai, MD, PhD^4,5^ David Dowdy, PhD^1^

**Author affiliations:**

^1^ Department of Epidemiology, Johns Hopkins Bloomberg School of Public Health, Baltimore, MD 21205, USA

^2^ Division of Infectious Disease, Johns Hopkins University School of Medicine, Baltimore, MD 21205, USA

^3^ Department of Global Health and Development, London School of Hygiene and Tropical Medicine, London WC1E 7HT, UK

^4^ Department of Epidemiology & Biostatistics & McGill International TB Centre, McGill University, Montreal, QC H3A 1A2, Canada

^5^ Manipal McGill Centre for Infectious Diseases, Manipal Academy of Higher Education, Manipal, India

Contents

[1. Cost Analysis 3](#_Toc12877754)

[1.1. An overview of the 2025 National Strategic Plan (NSP) for Xpert scale-up in India 3](#_Toc12877755)

[1.2. Index per-test unit cost for laboratories 4](#_Toc12877756)

[1.3. Distribution of daily test volume and calculating mean per-test cost 6](#_Toc12877757)

[1.4. Calculating sample transport costs 9](#_Toc12877758)

[1.5. Calculating the cost difference between decentralized Xpert (DXP) and centralized Xpert (CXP) 11](#_Toc12877759)

[1.6. Sensitivity analysis of GeneXpert equipment useful life – a worked example 17](#_Toc12877760)

[2. The simulation model 20](#_Toc12877761)

[2.1. Underlying simulation model 20](#_Toc12877762)

[2.2. TB treatment decision and Xpert performance 22](#_Toc12877763)

[2.3. Experimental framework for studying the impact of Xpert 23](#_Toc12877764)

[2.4. Simulation process 23](#_Toc12877765)

[2.5. Model calibration process 23](#_Toc12877766)

[2.6. Summary of model parameters 26](#_Toc12877767)

[3. Sensitivity analysis 28](#_Toc12877768)

[3.1. One-way sensitivity analyses 28](#_Toc12877769)

[3.2. Sensitivity and threshold analysis to incremental pre-treatment loss to follow up in the centralized scenario 29](#_Toc12877770)

[3.3. Sensitivity analysis to role of smear 32](#_Toc12877771)

# Cost Analysis

## An overview of the 2025 National Strategic Plan (NSP) for Xpert scale-up in India

India’s 2025 National Strategic Plan for laboratory systems and diagnosis is developed around the goal of achieving universal access to early and accurate diagnosis of TB. One of the key activities within the laboratory strengthening program is to roll-out cartridge-based nucleic acid amplification test (CBNAAT – Xpert MTB/RIF) throughout the country, built upon the Revised National Tuberculosis Control Programme (RNTCP)’s initial success in implementing more than 600 cartridge-based nucleic acid amplification tests (CBNAAT) between 2012 and 2017. The 2025 NSP aims to accelerate this process by making CBNAAT available to 8335 laboratory facilities. Table S1 summarizes expected national coverage and average testing volumes for CBNAAT based on the projected numbers reported in the 2025 NSP.

| **Indicators** | **Year** | | | | | | | | | | |
| --- | --- | --- | --- | --- | --- | --- | --- | --- | --- | --- | --- |
|  | **2015** | **2016** | **2017** | **2018** | **2019** | **2020** | **2021** | **2022** | **2023** | **2024** | **2025** |
| Total # of diagnostic tests* | 9,343,004 | 9,722,000 | 10,718,000 | 12,330,000 | 14,565,000 | 15,960,000 | 19,300,000 | 20,390,000 | 21,205,000 | 21,270,000 | 21,495,000 |
| # of Xpert Labs | 123 | 628 | 1235 | 2235 | 3235 | 5235 | 6235 | 6985 | 7585 | 8085 | 8335 |
| Coverage @ DTC** | 17% | 86% | 100% | 100% | 100% | 100% | 100% | 100% | 100% | 100% | 100% |
| Coverage @ TBU | 0% | 0% | 11% | 33% | 55% | 99% | 100% | 100% | 100% | 100% | 100% |
| Coverage @ DMC (staggered) | 0.00% | 0.00% | 0.00% | 0.00% | 0.00% | 0.00% | 7% | 12.32% | 16.64% | 20.23% | 22.03% |
| Coverage @ DMC (immediate)** | 0% | 0% | 4% | 11% | 18% | 32% | 40% | 45% | 49% | 53% | 55% |
| Total # Xpert tests performed @ DTC | 9,343,004 | 9,722,000 | 10,329,600 | 10,996,644 | 11,942,714 | 10,791,475 | 11,662,151 | 11,221,244 | 10,754,966 | 10,023,266 | 9,742,916 |
| Total # Xpert tests performed @ DMC | 0 | 0 | 388,400 | 1,333,356 | 2,622,286 | 5,168,525 | 7,637,849 | 9,168,756 | 10,450,034 | 11,246,734 | 11,752,084 |
| Total # DMC w/ XP | 0 | 0 | 504 | 1504 | 2504 | 4504 | 5504 | 6254 | 6854 | 7354 | 7604 |
| Annual average # of test / DTC | 12,781 | 13,300 | 14,131 | 15,043 | 16,338 | 14,763 | 15,954 | 15,351 | 14,713 | 13,712 | 13,328 |
| Daily average # of XP / DTC | 51 | 53 | 57 | 60 | 65 | 59 | 64 | 61 | 59 | 55 | 53 |
| Annual average # of test / DMC | 0 | 0 | 771 | 887 | 1,047 | 1,148 | 1,388 | 1,466 | 1,525 | 1,529 | 1,546 |
| Daily average # of test / DMC  (250 d/yr) | 0 | 0 | 3 | 4 | 4 | 5 | 6 | 6 | 6 | 6 | 6 |

**Table S1.** The projection of Xpert testing coverage and testing volumes based on the National Strategic Plan (NSP) for Tuberculosis Elimination 2017-2025

* total number of diagnostic tests is estimated based on the projected total number of Xpert and smear tests performed in the 2025 NSP report

** Xpert test coverage (%) is based on: 1) prioritizing Xpert scale-up at central locations (DTCs), and 2) total number of DTCs (n=731) and DMCs (n=13,908)

## Index per-test unit cost for laboratories

Index per-test unit cost of Xpert was calculated based on data derived from previous costing studies conducted as part of the Foundation for Innovative New Diagnostics diagnostic field studies in India (all unit prices and unit cost data), Vietnam and Malawi,[1] and from previous published work by Vassall et al[2, 3] using a bottom-up micro-costing method based on the activity based costing (ABC) framework. Xpert per-test cost for both centralized and decentralized Xpert include costs associated with unused equipment capacity based on the equipment daily capacity maximum (EDC_max_) of the key laboratory equipment. For a four module GeneXpert IV system, we assumed EDC_max_ to be 16 tests for a laboratory staffed to operate 8 hours per day. For one GeneXpert Omni (single module), we assumed a total of four tests can be performed under the same operating condition. Therefore, any number of tests performed below EDC_max_ in a given day would result in incorporation of costs associated with unused GeneXpert system modules. For example, if a laboratory performed 10 Xpert tests, a total of 6 individual GX4 slots were unused that day. Thus, six per-module costs would be incurred as additional cost to this day as costs associated with unused capacity. This process was repeated for the decentralized Xpert with relevant estimated pricing[4] and needed capacity to meet a 90% same-day testing requirement (Table S2). Prices of GeneXpert machines and cartridges were based on concessionary pricing[5] with additional 20% added to reflect costs for procurement (import taxes, shipping, and other charges) and installation.

We estimated a “per-slot cost”, defined as the fixed component of the Xpert equipment cost per two-hour time slot (i.e., one module for two hours during business hours) that is necessary to perform an Xpert test and report results. To calculate this per-slot cost, we assume in the base case that the useful life of a module was not linked to the frequency of using that module for Xpert testing (i.e. higher testing volume has no association with the differences in the module’s duration of useful life). We assumed a useful life of 10-15 years (10 years for peripheral level use, 15 years for centralized testing), but varied both the useful life and the linkage between useful life and module use in sensitivity analyses in Section 1.6.

We estimated the per-module cost as DC/EDC_max_ where DC is daily cost of the GeneXpert equipment and EDC_max_ is the maximum daily capacity (i.e., four two-hour time slots per module, so 4 * number of modules) . DC was calculated based on GeneXpert equipment price (P), marked up by 20% to reflect procurement costs and annual cost of maintenance service (AMC) plan using the following two equations:

1. DC = (P_GeneXpert system_ / AF) + AMC, where
2. *AF = (1-DF)/r, where DF = 1/(1+r)^n^, n = expected-useful life years (ELY) and r = discount rate*

As such, a GeneXpert IV system would have an estimated annual cost of $4039. This is calculated based on an annualized total equipment procurement cost of $1759 ($21,000 of equipment procurement price [using FIND negotiated pricing and added 20% procurement cost] divided by AF (11.93 calculated based on the equation #2, using discount rate of 3% and 15 ELY) and $2280 in annual maintenance cost (FIND negotiated 3-year service warranty contract of $6840). For a single-module GeneXpert device (Edge or Omni), the annual cost of $1157 reflects an annualized equipment procurement cost of $407 (procurement cost of $3474 – calculated based on an estimated equipment price of $2895 and 20% procurement cost – divided by AF of 8.53 calculated based on 3% discount rate and 10 ELY) and an annual maintenance cost of $750 (estimated based on 3 years warranty extension cost of a 2-module GeneXpert system, currently valued at $4500).

From these costs, we assessed the daily costs of owning respective systems at central vs. peripheral laboratories based on each laboratory operating 250 days per year ($4039/250 = $16.2 for GeneXpert IV and $1157/250 = $4.6 for single-module GeneXpert). After dividing by EDC_max_ (16 tests per day using GeneXpert IV and four tests per day using single-module GeneXpert), the GeneXpert IV system and single-module GeneXpert would have daily costs per test slot of $1.01 ($16.2/16) and $1.15 ($4.6/4) respectively.

| # of samples per batch processed | # of Omni/Edge modules | | | |
| --- | --- | --- | --- | --- |
|  | 1 | 2 | 3 | 4 |
| 0 | 4.6 | 9.2 | 13.8 | 18.4 |
| 1 | 18.55 | 23.15 | 27.75 | 32.35 |
| 2 | 15.31 | 19.91 | 24.51 | 29.11 |
| 4 | 13.70 | 18.30 | 22.90 | 27.50 |
| 6 | 13.91 | 16.21 | 20.81 | 25.41 |
| 7 | 13.93 | 15.08 | 19.68 | 24.28 |
| 10 | 13.63 | 18.27 | 15.93 | 20.53 |
| 14 | 13.51 | 17.34 | 22.79 | 15.93 |
| 16 | 13.33 | 16.76 | 20.99 | 13.33 |

| # of samples per batch processed | # of GX4 modules | | |
| --- | --- | --- | --- |
|  | 1 GX4 | 2 GX4 | 3 GX4 |
| 0 | 16.14 | 32.29 | 48.43 |
| 1 | 32.14 | 63.42 | 110.84 |
| 2 | 22.12 | 52.39 | 98.80 |
| 4 | 17.10 | 45.35 | 89.75 |
| 6 | 15.78 | 42.02 | 84.39 |
| 7 | 15.57 | 40.79 | 82.16 |
| 10 | 14.51 | 36.71 | 75.05 |
| 14 | 13.97 | 32.13 | 66.44 |
| 16 | 13.74 | 29.88 | 62.17 |
| … | | | |
| 32 | 13.74 | 13.74 | 29.88 |
| … | | | |
| 48 | 13.74 | 13.74 | 13.74 |

**Table S2.** Index per-test unit cost of Xpert by varied workloads and used of GeneXpert platform (Omni/Edge vs. GX4)

## Distribution of daily test volume and calculating mean per-test cost

To assess annual average per-test cost based on dynamic daily test volumes at DTC and DMC laboratories, we generated a set of 250 unique random numbers (representing 1 year of laboratory operations) for each laboratory representing average daily workload (λ = lambda) using a Poisson distribution. In our modeling work, we evaluated scenarios of λ ranging between 0.1 to 10 for DMC and between 10 and 70 for DTC where each λ scenario represents a unique laboratory. Based on the distribution of the daily workloads, we then calculated the number of GeneXpert platform (Omni/Edge or GX4) modules required to ensure 90% same-day (decentralized) and two-day (centralized) turn-around. Then, for each random number (representing number of patient samples tested for a given day), we assigned the corresponding index per-test cost of Xpert referenced from Table S1 (for example, if a random number generated was 4, we assigned index a per-test unit cost that represented a mean workload of 4 tests for decentralized or centralized testing in that simulated facility). Average per-test cost for each laboratory was calculated based on the total cost (inclusive of annual external quality control – EQA with 3 panel testing and shipping cost of the panels)^[[1]](#footnote-1)^ divided by the total number of tests performed in the set of 250 independent per-test cost estimates. Full results are shown in Table S3 and S4.

| **Indicators** | **λ 0.1** | **λ 0.2** | **λ 0.3** | **λ 0.5** | **λ 0.7** | **λ 1** | **λ 2** | **λ 3** | **λ 4** | **λ 5** | **λ 6** | **λ 7** | **λ 8** | **λ 9** | **λ 10** |
| --- | --- | --- | --- | --- | --- | --- | --- | --- | --- | --- | --- | --- | --- | --- | --- |
|  | *Test volume statistics* | | | | | | | | | | | | | | |
| Average # Samples / Day | 0.1 | 0.2 | 0.3 | 0.5 | 0.7 | 1.1 | 1.9 | 3 | 3.9 | 5.1 | 5.8 | 7 | 7.9 | 8.9 | 10.2 |
| *Highest daily workload* | 1 | 2 | 2 | 2 | 3 | 4 | 6 | 7 | 9 | 10 | 11 | 13 | 13 | 16 | 18 |
| *Lowest daily workload* | 0 | 0 | 0 | 0 | 0 | 0 | 0 | 0 | 0 | 1 | 2 | 2 | 3 | 3 | 4 |
| Total # of patients tested (annual) | 27 | 51 | 64 | 121 | 163 | 263 | 469 | 752 | 973 | 1277 | 1456 | 1739 | 1986 | 2234 | 2554 |
| *Range (Low)* | 0 | 40 | 58.5 | 93.5 | 143 | 214 | 367 | 611.5 | 863.5 | 1126 | 1367.5 | 1598.5 | 1863.5 | 2111 | 2395 |
| *Range (High)* | 39 | 57.5 | 92.5 | 142 | 213 | 366 | 610.5 | 862.5 | 1125 | 1366.5 | 1597.5 | 1862.5 | 2110 | 2394 |  |
|  | *Calculating # of Omni modules required (90% same day turn-around guarantee)* | | | | | | | | | | | | | | |
| Frequency beyond 4 tests per day | 0 | 0 | 0 | 0 | 0 | 0 | 10 | 41 | 83 | 150 | 173 | 227 | 241 | 238 | 247 |
| Frequency beyond 8 tests per day | 0 | 0 | 0 | 0 | 0 | 0 | 0 | 1 | 7 | 14 | 35 | 66 | 100 | 127 | 175 |
| Frequency beyond 12 tests per day | 0 | 0 | 0 | 0 | 0 | 0 | 0 | 0 | 0 | 0 | 0 | 6 | 12 | 44 | 53 |
| % same day coverage with 1 Omni/Edge | 100% | 100% | 100% | 100% | 100% | 100% | 96% | 84% | 67% | 40% | 31% | 9% | 4% | 5% | 1% |
| % same day coverage with 2 Omni/Edge | NA | NA | NA | NA | NA | NA | NA | 100% | 97% | 94% | 86% | 74% | 60% | 49% | 30% |
| % same day coverage with 3 Omni/Edge | NA | NA | NA | NA | NA | NA | NA | NA | NA | NA | 100% | 98% | 95% | 82% | 79% |
| # of Omni Units Required | 1 | 1 | 1 | 1 | 1 | 1 | 1 | 2 | 2 | 2 | 3 | 3 | 3 | 4 | 4 |
|  | *Cost Estimates* | | | | | | | | | | | | | | |
| Annual EQA cost (PE) | $115 | $115 | $115 | $115 | $115 | $115 | $115 | $231 | $231 | $231 | $346 | $346 | $346 | $461 | $461 |
| Total Cost (PE) | $1,642 | $1,967 | $2,147 | $2,916 | $3,482 | $4,804 | $7,496 | $14,394 | $17,694 | $21,971 | $30,085 | $34,353 | $37,704 | $48,910 | $52,892 |
| % EQA | 7% | 6% | 5% | 4% | 3% | 2% | 2% | 2% | 1% | 1% | 1% | 1% | 1% | 1% | 1% |
| Average Cost / Test | $60.81 | $38.58 | $33.55 | $24.10 | $21.36 | $17.83 | $15.98 | $19.14 | $18.19 | $17.20 | $20.66 | $19.75 | $18.98 | $21.89 | $20.71 |

**Table S3.** A summary table of assumptions used in decentralized Xpert (DXP) using GeneXpert Omni/Edge and average per-test results for district microscopy centers with different daily workloads.

| **Indicators** | **DTC (10)** | **DTC (20)** | **DTC (30)** | **DTC (40)** | **DTC (50)** | **DTC (60)** | **DTC ( > 70)** |
| --- | --- | --- | --- | --- | --- | --- | --- |
|  | *Test volume statistics* | | | | | | |
| Average # of samples / day | 9.90 | 19.83 | 29.61 | 39.71 | 50.23 | 59.61 | 69.64 |
| Highest daily workload | 22 | 36 | 50 | 60 | 71 | 85 | 88 |
| Lowest daily workload | 3 | 10 | 17 | 23 | 33 | 36 | 49 |
| Total # of patients tested | 2,475 | 4,957 | 7,403 | 9,928 | 12,557 | 14,903 | 17,411 |
| *Range (Low)* | 0 | 3,715 | 6,179 | 8,665 | 11,242 | 13,729 | 16,156 |
| *Range (High)* | 3,716 | 6,180 | 8,666 | 11,243 | 13,730 | 16,157 | 20,000 |
|  | *Calculating # of Omni modules required (90% same day turn-around guarantee)* | | | | | | |
| Frequency beyond 32 test per day | 0 | 2 | 61 | 224 | 250 | 250 | 250 |
| Frequency beyond 48 test per day | 0 | 0 | 1 | 14 | 146 | 146 | 146 |
| Frequency beyond 64 test per day | 0 | 0 | 0 | 0 | 5 | 5 | 5 |
| % two-day test coverage w/ 1 GX4 | 100% | 99% | 76% | 10% | 0% | 0% | 0% |
| % two day test coverage w/ 2 GX4 | 100% | 100% | 100% | 94% | 42% | 42% | 42% |
| % two day test coverage w/ 3 GX4 | 100% | 100% | 100% | 100% | 98% | 98% | 98% |
| # of GX 4 Unites required | 1 | 1 | 2 | 2 | 3 | 3 | 3 |
|  | *Cost Estimates* | | | | | | |
| Annual EQA cost | $461 | $461 | $922 | $922 | $1,383 | $1,383 | $1,383 |
| Total Cost | $37,111 | $68,768 | $124,096 | $139,272 | $190,753 | $208,535 | $240,533 |
| % EQA | 1% | 1% | 1% | 1% | 1% | 1% | 1% |
| Average Cost / Test | $14.99 | $13.87 | $16.76 | $14.03 | $15.19 | $13.99 | $13.82 |

**Table S4.** A summary table of assumptions used in centralized Xpert using GX4 and average per-test results for district TB centers (DTC) with different mean daily workloads (not including sample transport cost) in parentheses.

## Calculating sample transport costs

To assess the complete centralized Xpert testing costs inclusive of sample transport, we first assessed the Indian TB health system and test volumes under the 2015 NSP to develop four key variables to determine transport costs (Table S5): average number of DMCs per DTC, average distance between facilities, range of number of samples from DMC (0.1 to 12 samples per clinic), and % TB sample volume per sample transport (ranging between 5% to 100%, where the percentage indicates exclusiveness of sample transport network for TB).[6, 7] Key cost parameters – direct (overhead) and indirect (fuel, HR, materials) – were derived from a TB patient outreach by community health workers in rural India conducted by one of the authors.[8]

**Table S5.** Scenarios and cost parameters used for Simulated Specimen Transport Networks in India.

For each DMC sample category (number of samples sent from a DMC – 5 unique scenarios of 3, 10, 18, 27, and 39 DMCs per DTC), a total of 100 possible sample transport cost combinations were created based on independent variation of three key variables: distance (5 categories), number of patient samples (5 categories) and percent TB volume per transport (4 categories^[[2]](#footnote-2)^). For each combination, we calculated the total variable and fixed cost (total fixed cost per day + total variable costs) for four scenarios of sample transport percentage devoted to Xpert (5, 10, 50, and 100%). This process was repeated for remining 4 DMC sample categories. One complete set of transport calculations (for DMC sample category 3) is shown in Table S6.

A complete overview (a total of 500 cost sets) and trend of per-sample transport cost for each DTC (λ) based on transport sample volume is shown in Figure S1. Of these, we eliminated scenarios that carried more than 100 TB samples (sputum) and total travel distance greater than 250 km/day as these scenarios are highly unlikely in the Indian setting (260 sets). Remaining scenarios were then categorized based on the number of TB samples transported (we repeated this exercise for minimum, inter-quartile ranges (1Q, median, 3Q), and maximum of sample transport costs).

Mean per-sample costs were individually matched to the daily test volumes from the DTC (λ) distribution set for relevant DTC scenarios (10 to >70). For example, if DTC 20 (center tests a mean of 20 tests per day) on day 10 of the 250 daily test volume sets indicate 16 samples (16 tests performed), we matched the mean sample transport cost according to the sample transport definition range shown in Table S7. Thus, observation included unique Xpert testing cost and sample transport costs, and these were summed (also with annual EQA costs) across 250 data points for each DTC to compute total annual cost. These were then divided by the total number of patient samples tested across the 250 samples for each DTC (λ) to calculate mean per-test cost in the centralized testing scenario. A graphic illustration of the step-by-step process of assessing unit costs inclusive of sample transport costs is shown in Figure S2, and a sample full summary of mean per-test cost is shown in Table S8 (used to calculate the cost difference between centralized and decentralized testing).

## Calculating the cost difference between decentralized Xpert (DXP) and centralized Xpert (CXP)

We used Table S3 (mean DMC Xpert per-test costs) and Table S8 (mean DTC Xpert per-test costs, inclusive of sample transport) to compute unit cost difference between the DXP and CXP scenarios. This was assessed based three main components: 1) DMC testing volume (range between 0.1 to 10 samples per-day – a total of 15 categories), 2) DTC testing volumes (between 10 to 70 or more per day – a total of 7 categories), and 3) exclusivity of TB sample in sample transport operations (between 5 and 100% - a total of 4 categories). We represent this using heat map analysis of a combined 15 sets of cost difference comparisons between varied levels of DTC testing volume and the exclusivity of TB sample in sample transport operations (Figure S3). Red represents cost difference favoring CXP (DMC Xpert per-test costs higher than that of the DTC) whereas blue represents costs favoring DXP (DMC Xpert per-test costs lower than that of the DTC).

| **Scenario Combinations** | **# of Clinics visited (1)** | **Total Distance (2)** | **Average # of Samples (3)** | **Total Variable Cost (4)** | **Total Cost** | | | | **Cost / Specimen transported** | | | |
| --- | --- | --- | --- | --- | --- | --- | --- | --- | --- | --- | --- | --- |
|  |  |  |  |  | % TB sample volume (5-100%) | | | | | | | |
|  |  |  |  |  | 5% | 10% | 50% | 100% | 5% | 10% | 50% | 100% |
| 11 | 3 | 15.33 | 0.35 | 32.81 | 5.59 | 11.17 | 55.85 | 111.70 | 16.13 | 32.26 | 161.32 | 322.63 |
| 21 | 3 | 30.30 | 0.35 | 64.84 | 7.19 | 14.37 | 71.87 | 143.73 | 20.76 | 41.51 | 207.57 | 415.14 |
| 31 | 3 | 39.22 | 0.35 | 83.93 | 8.14 | 16.28 | 81.41 | 162.83 | 23.52 | 47.03 | 235.15 | 470.31 |
| 41 | 3 | 48.74 | 0.35 | 104.30 | 9.16 | 18.32 | 91.60 | 183.19 | 26.46 | 52.91 | 264.56 | 529.12 |
| 51 | 3 | 90.38 | 0.35 | 193.41 | 13.62 | 27.23 | 136.15 | 272.30 | 39.33 | 78.65 | 393.25 | 786.51 |
| 12 | 3 | 15.33 | 10.39 | 32.81 | 5.59 | 11.17 | 55.85 | 111.70 | 0.54 | 1.08 | 5.38 | 10.75 |
| 22 | 3 | 30.30 | 10.39 | 64.84 | 7.19 | 14.37 | 71.87 | 143.73 | 0.69 | 1.38 | 6.92 | 13.84 |
| 32 | 3 | 39.22 | 10.39 | 83.93 | 8.14 | 16.28 | 81.41 | 162.83 | 0.78 | 1.57 | 7.84 | 15.68 |
| 42 | 3 | 48.74 | 10.39 | 104.30 | 9.16 | 18.32 | 91.60 | 183.19 | 0.88 | 1.76 | 8.82 | 17.64 |
| 52 | 3 | 90.38 | 10.39 | 193.41 | 13.62 | 27.23 | 136.15 | 272.30 | 1.31 | 2.62 | 13.11 | 26.22 |
| 13 | 3 | 15.33 | 13.85 | 32.81 | 5.59 | 11.17 | 55.85 | 111.70 | 0.40 | 0.81 | 4.03 | 8.07 |
| 23 | 3 | 30.30 | 13.85 | 64.84 | 7.19 | 14.37 | 71.87 | 143.73 | 0.52 | 1.04 | 5.19 | 10.38 |
| 33 | 3 | 39.22 | 13.85 | 83.93 | 8.14 | 16.28 | 81.41 | 162.83 | 0.59 | 1.18 | 5.88 | 11.76 |
| 43 | 3 | 48.74 | 13.85 | 104.30 | 9.16 | 18.32 | 91.60 | 183.19 | 0.66 | 1.32 | 6.61 | 13.23 |
| 53 | 3 | 90.38 | 13.85 | 193.41 | 13.62 | 27.23 | 136.15 | 272.30 | 0.98 | 1.97 | 9.83 | 19.66 |
| 14 | 3 | 15.33 | 20.77 | 32.81 | 5.59 | 11.17 | 55.85 | 111.70 | 0.27 | 0.54 | 2.69 | 5.38 |
| 24 | 3 | 30.30 | 20.77 | 64.84 | 7.19 | 14.37 | 71.87 | 143.73 | 0.35 | 0.69 | 3.46 | 6.92 |
| 34 | 3 | 39.22 | 20.77 | 83.93 | 8.14 | 16.28 | 81.41 | 162.83 | 0.39 | 0.78 | 3.92 | 7.84 |
| 44 | 3 | 48.74 | 20.77 | 104.30 | 9.16 | 18.32 | 91.60 | 183.19 | 0.44 | 0.88 | 4.41 | 8.82 |
| 54 | 3 | 90.38 | 20.77 | 193.41 | 13.62 | 27.23 | 136.15 | 272.30 | 0.66 | 1.31 | 6.55 | 13.11 |
| 15 | 3 | 15.33 | 41.55 | 32.81 | 5.59 | 11.17 | 55.85 | 111.70 | 0.13 | 0.27 | 1.34 | 2.69 |
| 25 | 3 | 30.30 | 41.55 | 64.84 | 7.19 | 14.37 | 71.87 | 143.73 | 0.17 | 0.35 | 1.73 | 3.46 |
| 35 | 3 | 39.22 | 41.55 | 83.93 | 8.14 | 16.28 | 81.41 | 162.83 | 0.20 | 0.39 | 1.96 | 3.92 |
| 45 | 3 | 48.74 | 41.55 | 104.30 | 9.16 | 18.32 | 91.60 | 183.19 | 0.22 | 0.44 | 2.20 | 4.41 |
| 55 | 3 | 90.38 | 41.55 | 193.41 | 13.62 | 27.23 | 136.15 | 272.30 | 0.33 | 0.66 | 3.28 | 6.55 |

**Table S6.** Sample transport scenarios and calculation of per-sample cost for TB samples collected at 3 peripheral centers

| **Definition Range** *(# of sample transported)* | | **Category assigned** | **DTC (λ)** | **Mean** | | | |
| --- | --- | --- | --- | --- | --- | --- | --- |
|  |  |  |  | 5% | 10% | 50% | 100% |
| 1 or less | | 1 | DTC (10) | 20.56 | 41.12 | 205.58 | 411.16 |
| 2 | | 2 | DTC (10) | 10.31 | 20.62 | 103.10 | 206.21 |
| 3 | | 3 | DTC (10) | 7.83 | 15.67 | 78.33 | 156.66 |
| 4 | | 4 | DTC (10) | 5.58 | 11.16 | 55.78 | 111.57 |
| 5 | to 10 | 5 | DTC (10) | 0.84 | 1.68 | 8.41 | 16.82 |
| 10 | to 15 | 6 | DTC (20) | 0.63 | 1.26 | 6.31 | 12.62 |
| 15 | to 20 | 7 | DTC (20) | 0.42 | 0.84 | 4.21 | 8.41 |
| 20 | to 30 | 8 | DTC (30) | 0.53 | 1.06 | 5.29 | 10.59 |
| 30 | to 40 | 9 | DTC (30) | 0.30 | 0.61 | 3.04 | 6.07 |
| 40 | to 50 | 10 | DTC (40) | 0.34 | 0.69 | 3.44 | 6.87 |
| 50 | to 60 | 11 | DTC (50) | 0.26 | 0.53 | 2.65 | 5.29 |
| 60 | to 70 | 12 | DTC (60) | 0.26 | 0.52 | 2.58 | 5.16 |
| 70 | to 90 | 13 | DTC (70) | 0.26 | 0.52 | 2.61 | 5.22 |

**Table S7.** Sample transport category definitions (expressed as # of sample transported) and mean per-sample cost

| **Criteria** | | **DTC (λ)** | | | | | | | |
| --- | --- | --- | --- | --- | --- | --- | --- | --- | --- |
|  |  | *DTC10* | *DTC20* | *DTC30* | *DTC40* | *DTC50* | *DTC60* | *DTC( > 70)* |  |
| **% TB sample transport** | *5%* | 15.98 | 14.29 | 17.60 | 14.36 | 15.68 | 14.24 | 14.00 |  |
|  | *10%* | 16.91 | 14.78 | 18.02 | 14.70 | 15.98 | 14.51 | 14.26 |  |
|  | *50%* | 24.32 | 18.67 | 21.38 | 17.36 | 18.38 | 16.66 | 16.35 |  |
|  | *100%* | 33.59 | 23.54 | 25.59 | 20.70 | 21.38 | 19.35 | 18.96 |  |

**Table S8.** A summary of mean centralized per-test cost including sample transport cost based on the daily testing volume at each DTC (λ) and % TB sample transported per transport

**Figure S1.** A complete graphic summary of per-sample transport cost for each for 260 likely sample transport scenarios, ordered by mean daily sputum sample transported to the DTC (x-axis) and exclusivity of the TB sample transported (in %)

Each line in the graph represents the exclusivity of TB sample transport (expressed in terms of percentage where 100% indicates sample transport network exclusively established to transport TB samples). All DTC daily test volume scenarios (between 10 to 70 or samples tested per day) and number of district microscopy centers (DMC, minimum 3 to maximum 39) were combined in this figure.


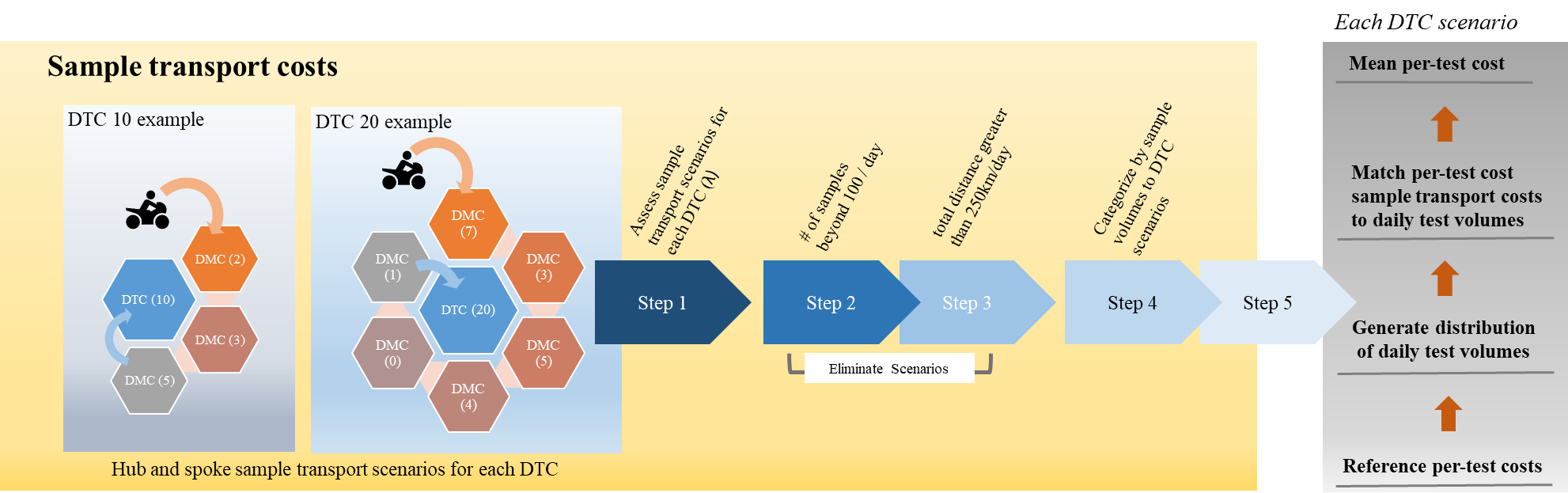


**Figure S2.** Complete process overview of unit cost calculation for each Xpert testing scenarios.

For each district TB center (DTC) volume scenarios, we assessed a range hub-and-spoke sample transport network arrangement based on the total distance traveled and the number of district microscopy centers (DMC) associated with each DTC (Step 1). Originally, a total of 500 possible sample transport scenarios were evaluated. We eliminated highly unlikely scenarios – scenarios with more than 100 samples transported per day in a single district TB center (DTC) region (Step 2) and total daily travel distance greater than 250km (Step 3) – based on India’s current TB health system structure. We then developed a reference table for sample transport cost based on total volume transported and relevant DTC categories (Step 4, Table 7). Then, we matched and assigned daily sample transport costs to the DTC daily test costs based on DTC daily test volumes (Step 5, see section 1.3) to compute mean DTC testing costs inclusive of sample transport.


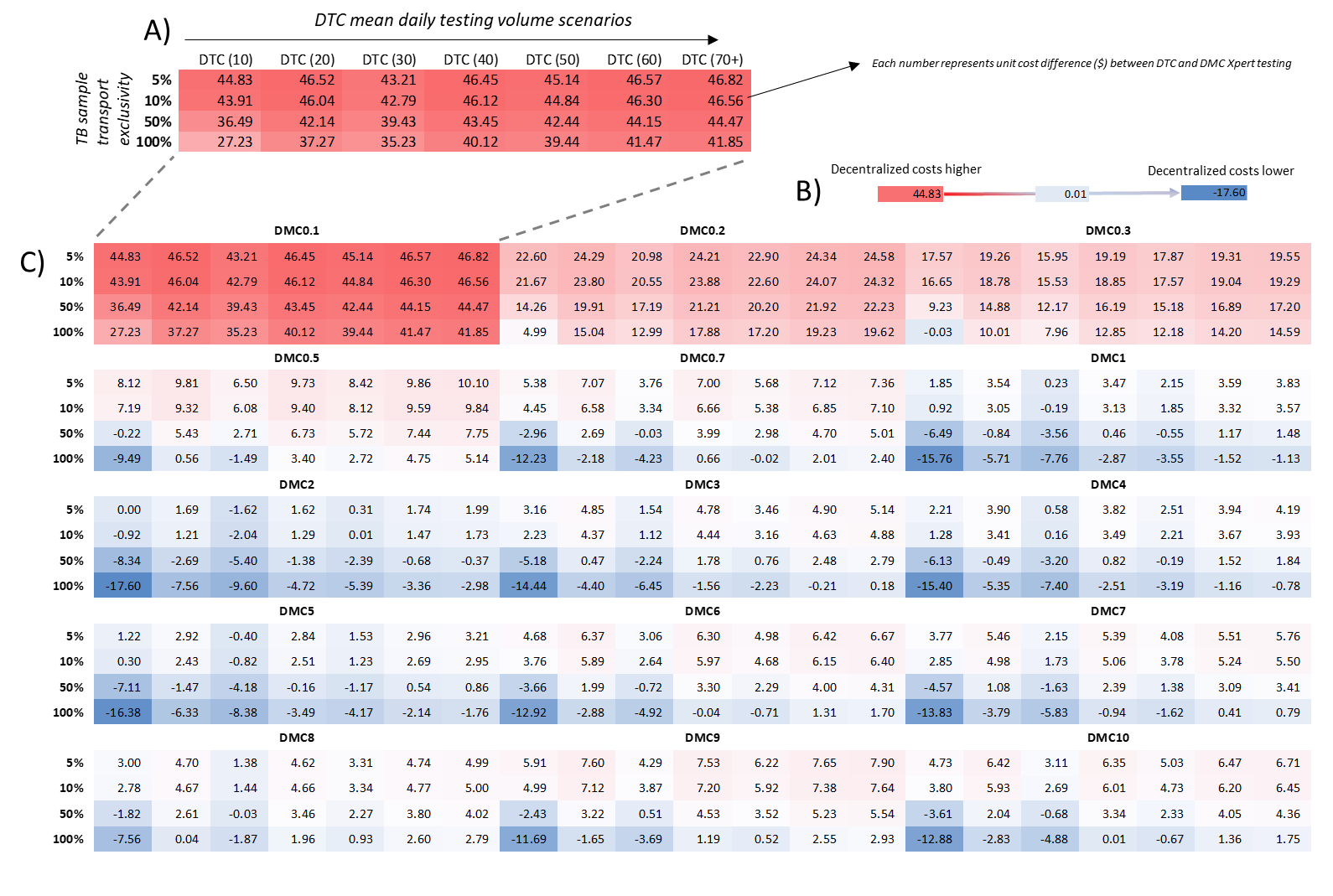


**Figure S3.** Illustrative summary of cost difference of decentralized Xpert testing (DMC) compared to centralized Xpert testing (DTC).

Each panel (e.g. Panel A) represents cost difference calculated for each decentralized Xpert testing scenario (presented by DMC volume – DMC 0.1 indicated DMC’s mean daily testing volume of 0.1) compared against varied levels of centralized testing volumes (DTC 10 to 70+) and the exclusivity of TB samples transported (5 to 100%). Red cells represent scenarios where decentralized Xpert testing would be more expensive than centralized testing whereas blue cells represent scenarios in which decentralized test costs would be favored over centralized testing, after accounting for sample transport costs (Panel B). Panel C is the complete illustrative overview of the cost differences of decentralized Xpert compared to centralized testing for all DMC, DTC, and exclusivity of TB sample transported scenarios.

## Sensitivity analysis of GeneXpert equipment useful life – a worked example

The annual cost of the GeneXpert system (IV or single-module) was assessed by combining annuitized equipment procurement costs (20% mark-up on equipment list price, then dividing this estimate by an annualization factor [AF]). AF is calculated based on the following equation:

*AF = (1-DF)/r, where DF = 1/(1+r)^n^, n = expected useful life (years) and r = discount rate*

For example, if we assume 2 years of equipment useful life years (ELY) under a 3% annual discount rate, AF will be 1.9135. We repeated this calculation for a total of four different ELY scenarios (2, 5, 10, and 15 years) relevant for the GeneXpert system where the table of AF is shown below:

| **Useful Life (years)** | **Annualization Factor** |
| --- | --- |
| 30 | 19.6004 |
| 15 | 11.9379 |
| 10 | 8.5302 |
| 5 | 4.5797 |
| 2 | 1.9135 |

**Table S9.** Annualization factor associated with relevant useful life assumed for capital assets.

Using this table, we provide an example of two MC test volume scenarios (0.3. and 3 per day) to demonstrate differences in mean per-test costs for four different ELY levels assumed for single-module GeneXpert (10 years of useful life was used for our main analysis). In doing so, we first repeated the steps provided in Section 1.2 to develop index per-test unit costs according to the number of GeneXpert devices procured and the number of tests performed at the MC level (Table S10, orange colored cells are primary estimates used in our main analysis). “0” tests represents a scenario in which the laboratory would not perform any test using the GeneXpert machine (i.e. no Xpert test performed) and the cost represents only the daily opportunity cost of ownership of the GeneXpert system. In the example below, the per-test unit cost of Xpert ranged between $4.16 to $20.52 depending on the number of GeneXpert modules procured and the estimated ELY.

| **# tests** | 1 GeneXpert Omni | | | | 2 GeneXpert Omni | | | |
| --- | --- | --- | --- | --- | --- | --- | --- | --- |
|  | **Estimated Useful Life (years)** | | | | **Estimated Useful Life (years)** | | | |
|  | *2* | *5* | *10* | *15* | *2* | *5* | *10* | *15* |
| 0 | $10.26 | $6.03 | $4.63 | $4.16 | $20.52 | $12.07 | $9.26 | $8.33 |
| 1 | $26.30 | $20.62 | $18.55 | $18.02 | $36.56 | $26.66 | $23.18 | $22.19 |
| 2 | $19.19 | $16.35 | $15.31 | $15.05 | $29.45 | $22.38 | $19.94 | $19.21 |
| 4 | $15.63 | $14.21 | $13.70 | $13.56 | $25.89 | $20.25 | $18.33 | $17.73 |
| 6 | $16.49 | $14.60 | $13.91 | $13.74 | $21.62 | $17.62 | $16.23 | $15.82 |
| 7 | $16.14 | $14.52 | $13.93 | $13.77 | $18.70 | $16.03 | $15.08 | $14.82 |
| 10 | $15.95 | $14.25 | $13.63 | $13.47 | $22.53 | $19.41 | $18.27 | $17.98 |
| 14 | $15.72 | $14.10 | $13.51 | $13.36 | $20.52 | $18.19 | $17.34 | $17.12 |
| 16 | $15.27 | $13.85 | $13.33 | $13.20 | $19.18 | $17.40 | $16.76 | $16.59 |

**Table S10.** Volume-specific index per-test unit cost (in 2015 United States Dollars) for GeneXpert Omni used in Microscopy Centers (MC) under different levels of equipment useful life (years).

Using Table S10 as a reference, we then repeated the steps specified in Section 1.3 to calculate mean per-test costs for the two MCs with mean daily testing volume of 0.3 and 3. These estimates do not account for costs associated with the quality assurance (QA) program that is uniformly implemented depending on the annual testing volume and not affected by ELY. Testing volume was the key driver of the per-test cost differences between the two MCs; however, the degree of this difference was significantly reduced with increasing ELY. Changes in the assumed useful life of Xpert had little impact on the estimated unit cost of Xpert when the useful life of Xpert was assumed to be long (10-15 years), as in this analysis. The assumed useful life of equipment also had greater influence in lower testing volume laboratories (λ=0.3), suggesting that unit cost of Xpert at lower volume laboratories is likely be more affected by issues of frequent equipment breakdown and replacement than higher volume laboratories (Table S11). Likewise, the degree of difference in the costs of centralized vs. decentralized testing will likely be greater than the estimates provided in our main analysis when the actual useful life years of GeneXpert equipment is lower than the 10 or 15 years assumed in our analysis. In such situations, cost-effectiveness estimates are likely to be more favorable to centralized testing (where equipment can be maintained for longer periods of time, with less variation in unit cost per test).

| Volume of MC testing | Assumed Equipment Useful Life Years (ELY) | | | |
| --- | --- | --- | --- | --- |
|  | 2 | 5 | 10 | 15 |
| MC (λ=0.3) | $52.48 | $35.12 | $29.21 | $27.37 |
| MC (λ=3) | $28.12 | $21.29 | $18.93 | $18.23 |
| % difference | 87% | 65% | 54% | 50% |

**Table S11.** Mean per-test cost of single-module Xpert (expressed in 2015 US dollars) at two microscopy centers with different average daily testing volumes and different assumptions about the useful life of Xpert equipment.

# The simulation model

Details regarding the underlying structure of our agent-based simulation model in terms of population demographics, contact network, TB natural history and TB care cascade are provided in our earlier publication,[9] and as such are not repeated here. In this section, we provide an overview of the new aspects of this model relevant to study of centralized vs. decentralized Xpert implementation.

## Underlying simulation model

Our baseline model simulated a self-contained population of 1,000,000 individuals corresponding to the catchment area of a single District TB Center (DTC). We modeled circulation of both drug-susceptible (DS) and rifampin-resistant (DR) TB strains in the population. The natural history of TB was modeled at an individual level (Figure S4), and the parameters were estimated from the literature (see Table S12 below).


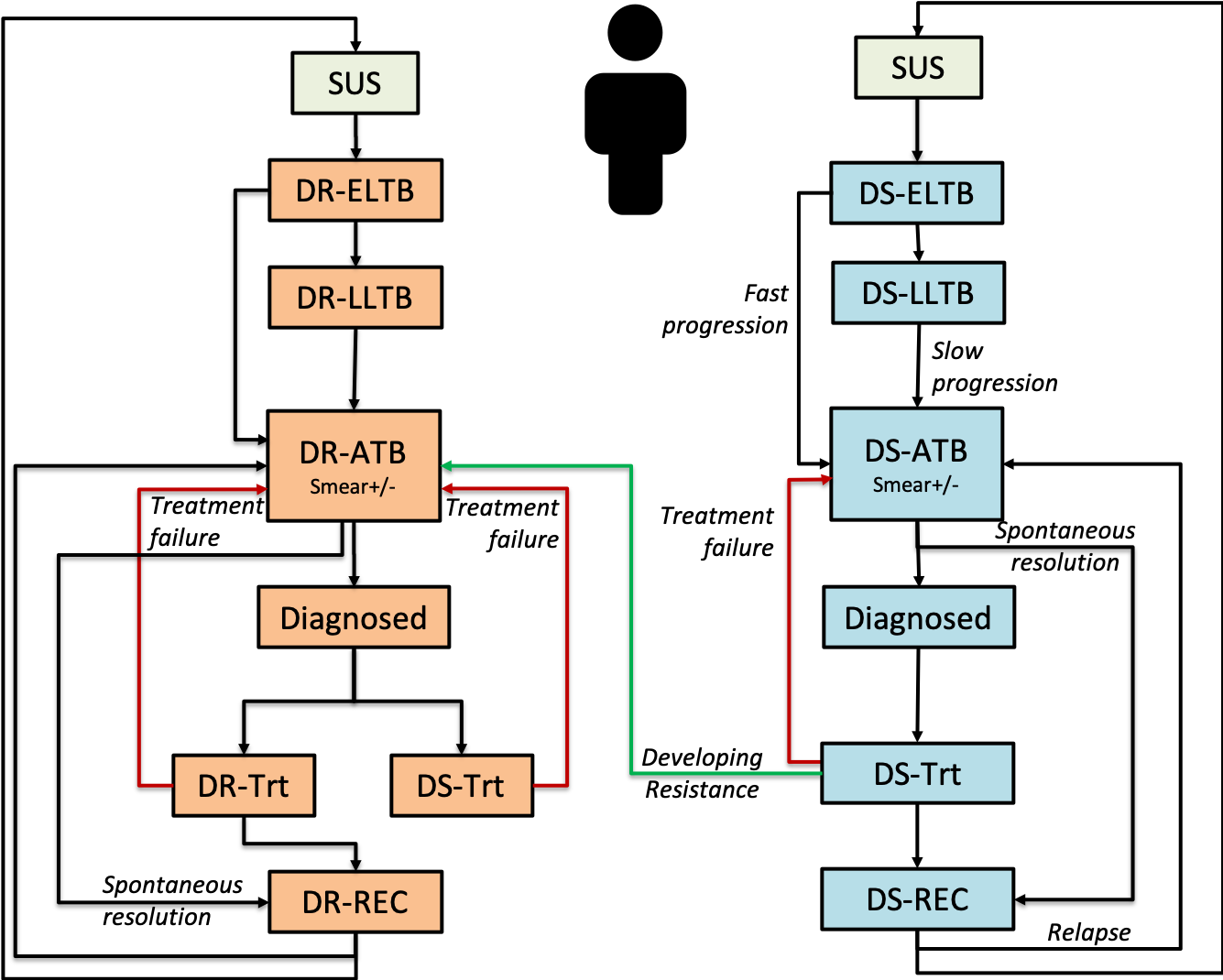


Figure S4: TB natural history outline. Upon successful transmission of TB, the infected individual enters the Early Latent TB (DS-/DR-ELTB) state for a period of five years (during which the per-time-step probability of active TB development is high but decreasing over time), followed by the Late Latent TB (DS-/DR-LLTB) state, which can last for many years and is associated with a lower, constant probability per time step (Slow Progression Rate) of developing active TB. Individuals with Active TB (DS-/DR-ATB) are symptomatic, infectious, and subject to increased mortality (each of which increases in degree over time after Active TB develops). DS-TB patients receiving DS-TB treatment are subject to a probability of treatment failure and acquiring drug resistance, upon which they will develop active DR-TB disease and will continue to transmit the DR strain to other susceptible individuals until treated. Latently infected or recovered individuals can get re-infected with either strain, at which time they move to the early latent stage (not shown).

Care-seeking behavior was modeled as a function of time since development of active TB disease. To represent the network of healthcare providers in a typical Indian setting, we considered 3 types of providers including 1) *informal providers* with no formal medical training; 2) *qualified private providers* with formal medical training but no access to microscopic labs; and 3) the *public sector* representing a designated microscopy center (DMC) ( Figure S5B). The DMC is a local health clinic with capacity for sputum smear microscopy and Xpert placement in the decentralized scenario. Following prior studies,[10–12] we estimated the probabilities of visits to each provider in this network.


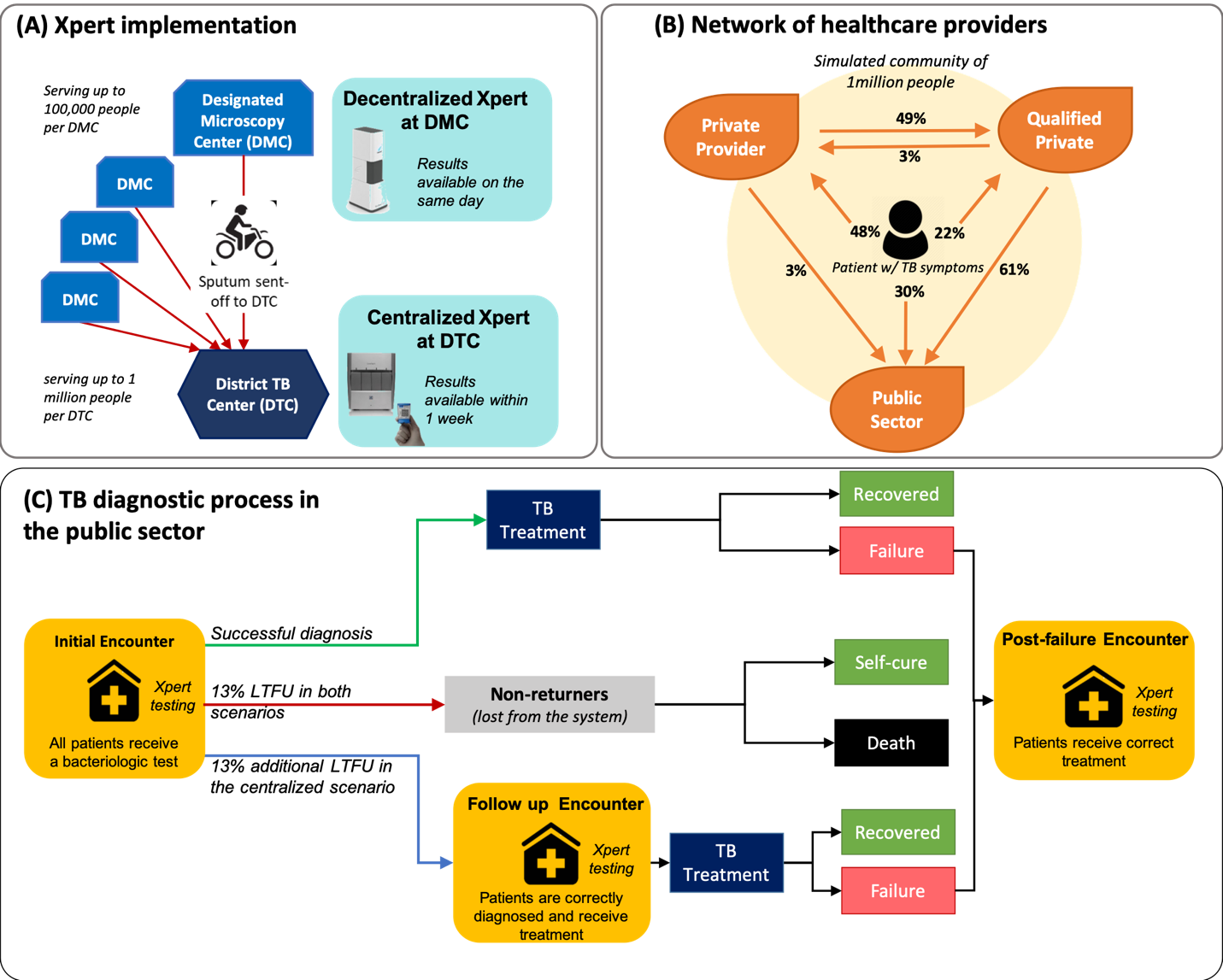


**Figure S5**: Schematic representation of the tuberculosis (TB) diagnostic system in India.

In modeling the TB diagnosis attempts via these providers, we made the following assumptions:

- A visit to informal-sector never leads to appropriate treatment of TB, and as such, the untreated patient will experience a probability for visiting other providers in the future.
- A proportion of patients visiting the private sector will be diagnosed and treated for DS-TB (estimated at 50%). However, such visits never lead to appropriate diagnosis/treatment of DR-TB.
- Visits to public sector – where a definitive bacteriologic diagnosis can be made – are modeled as a collection of “*encounters*”. An encounter represents all activities occurring from initial TB suspicion to arriving at a presumptive diagnosis, and may encompass multiple clinic visits.
- At the “*initial encounter*” in the public sector, all patients will receive a bacteriologic test to confirm TB diagnosis. At baseline (pre-Xpert era before year 2018), all patients are diagnosed via smear microscopy alone, but after implementation of Xpert (year 2018 to 2028), the initial diagnosis is made via Xpert as a standalone test (assuming no smear backup at baseline analysis).
- Upon TB diagnosis, patients can receive successful treatment or experience pre-treatment loss-to-follow-up due to diagnosis delays in the centralized scenario. In both groups, a proportion of patients (estimated at 13%) are considered to be permanently lost-to-follow-up. These individuals may undergo other diagnostic encounters, but those encounters do not result in TB diagnosis, and the cost of those encounters is not considered by the model. The conservative assumption of equal probability of permanent loss-to-follow-up between these two groups may underestimate the impact of Xpert in the decentralized scenario.
- Those experiencing pre-treatment loss-to-follow-up (but not permanently lost) can return for a future visit (“*follow-up encounter*”) in which Xpert is again performed and TB treatment is successfully initiated.
- Individuals receiving TB treatment who fail to recover will undergo a “*post-failure encounter”* , upon which they are tested via Xpert and will receive another course of correct TB treatment according to their underlying infection (DS-TB or DR-TB). Those experiencing more than one treatment failure will not return to care.
- In the absence of Xpert results (baseline model in the pre-Xpert era before year 2018), all newly diagnosed individuals (smear positive or those clinically diagnosed) received first-line TB treatment that lasts 6 months, with a treatment success rate in the public sector that is independent of where patients were referred from. We also assumed that diagnosis and treatment occur in the private sector and explicitly modeled patterns and outcomes of empiric treatment in the private sector when proper bacteriologic diagnosis referral to the public sector was not made.

## TB treatment decision and Xpert performance

In our model, TB patients were characterized by their underlying disease status that is differentiated by 1) New and Previous TB, and 2) DS and DR-TB disease, and they were further classified according to their smear and Xpert TB and RIF resistance detection status. As an individual’s TB disease becomes more advanced over time, they experience more symptoms and become more infectious. This will trigger an increase in care-seeking behavior as patients experience a higher burden of disease. Similarly, the smear status of patients (changing from smear negative to smear positive) will evolve with time since the onset of active disease, such that 70% of patients will be smear positive by 9 months since disease initiation. The sensitivity of Xpert for TB diagnosis is estimated at 67% among smear-negative patients and at 98% for smear positive individuals. We further assume that Xpert is 95% sensitive for detecting rifampin resistance among DR-TB patients. Specificity of Xpert for rifampin resistance was modeled as 98%. Finally, DS-TB therapy (a 6-month regimen) was assumed to have no impact on underlying DR-TB infection, while DR-TB therapy (a 20-month regimen) was assumed to clear any underlying DS-TB infections along with DR-TB.

## Experimental framework for studying the impact of Xpert

Our primary analysis was focused on the epidemiologic and economic impact of centralized vs. decentralized Xpert implementation. For both scenarios, we assumed that Xpert will be *intended* as an standalone test, replacing smear microscopy. As such, all patients presetting to the public sector (initial, follow up or post-failure encounter) will receive Xpert. While the cost of implementing Xpert in a centralized fashion (at the DTC level) may be lower than decentralized placement at local DMCs, the centralized testing is associated with a *pre-treatment loss-to-follow-up*. This is due to delays for transporting specimens from local DMCs to the central DTC in the centralized scenario. As such, the main impact of each scenario was determined based on the differential losses of patients who do not get proper diagnosis and initiation of TB treatment.

**Centralized Xpert scenario:** If Xpert is placed only at DTCs, we assumed that all patients presenting to DMCs with symptoms suggestive of TB will receive Xpert referral. Since patient samples are required to be transported from the DMCs to the central DTC for analysis, there is approximately a one-week delay for patients to receive Xpert results. We assume that this delay is associated with a 13% pre-treatment loss-to-follow-up after the initial encounter and vary this parameter in sensitivity analysis (see Section 3.1).

**Decentralized Xpert scenario :** In this scenario, we assumed that Xpert testing is performed with at least one battery operated Omni or Edge unit (procurement of the number of units optimized based on the demand at each DMC). Likewise, we assumed that all suspected TB patients (100%) will be tested with Xpert as an upfront test on the same day during the diagnostic visit. Decentralized Xpert is associated with no pre-treatment loss-to-follow-up.

## Simulation process

Our model is run in a timestep of one month, and outcomes are reported at the end of each simulated year. The core model simulates a population of 1 million people, representative of an average-size district in India that might contain a single DTC. For each Xpert testing scenario (centralized versus decentralized), we generated 2,000 independent simulations of TB transmission at the district level. To assess implementation at a city or state level, we then constructed larger simulated populations of 20 million people by combining 20 randomly sampled districts (with replacement) from the original pool of simulations. We repeated this procedure to achieve a sample of 2,000 larger-scale simulations with a population of 20 million individuals each. All outcomes were reported as median values with interquartile uncertainty ranges across these 2,000 larger-scale simulated populations.

## Model calibration process

In absence of DR-TB infection, the model was first brought to a DS-TB equilibrium, calibrated to estimated prevalence of DS-TB in India. Initial DR-TB infections were modeled through DS-TB treatment failures (leading to TB-drug resistance) at a time point of 40 years prior to the present day (i.e., year 1978), and were allowed to increase via new transmissions (assuming a lower relative infectiousness of DR-TB compared to DS-TB infections). Starting in year 2007, we modeled a gradual (linear) increase in availability of DR-treatment for patients who failed DS-treatment through the DOTS-Plus program in India, reaching all districts after six years of scale-up (approximately by year 2013). During this time, we assumed that TB diagnosis was entirely made by smear microscopy (no Xpert). The model was calibrated to 2017 TB epidemiological outcomes in India.[13] Using this calibrated model, we experimented with alternative strategies for implementation of Xpert testing in India (2018 to 2028). At the baseline analysis, we assumed that Xpert will be *intended* as a standalone test, replacing smear microscopy over the next decade (Figure S6).


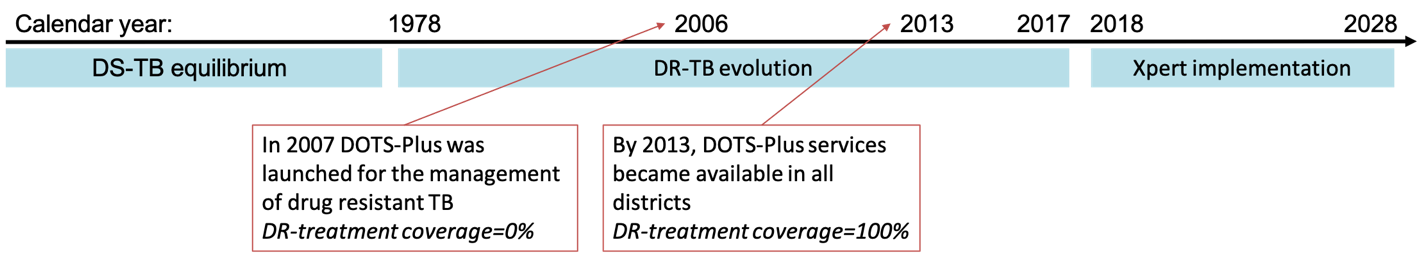


Figure S6: Simulation calibration process.

To calibrate the baseline model in year 2017, we tuned the unknown simulation parameters such that the DS-TB incidence, prevalence and mortality, as well as DR-TB incidence in year 2017, would match current estimates from the WHO and the associated burden of DR-TB among new and previously-treated cases at the time of diagnosis. Table S12 provides a list of all simulation parameters and calibration targets. Figure S7 provides a summary of model fit to calibration targets in year 2017.


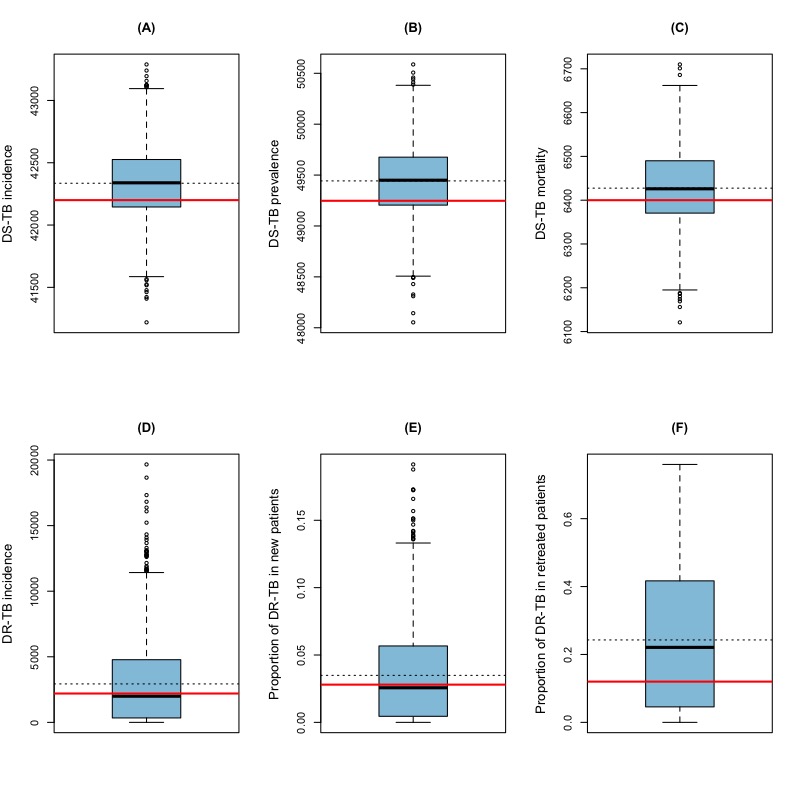


Figure S7: Model fit to calibration targets in year 2017. The blue box represents the upper and lower quartile of simulated outcomes (with median shown as solid black line and mean is shown as a dashed line), compared against calibration targets (red line) in year 2017. The whiskers mark the upper and lower 5% percentile of observations from 2,000 random simulations.

## Summary of model parameters

Table S12 provides a summary of all model parameters and calibration targets.

| Parameter | Value | Reference |
| --- | --- | --- |
| TB Natural History |  |  |
| Annual risk of progression from early latent state by age | (0 – 2] years: 0.20  (2 – 10] years: 0.02  (10 – 15] years: 0.06  (15 – 104] years: 0.09 | [14, 15] |
| Relative risk of progression from early latent state by year | Year1: 1  Year2: 0.41  Year3: 0.13  Year4: 0.086  Year5: 0.028 | [14] |
| Annual risk of LLTB progression | 5e-3 | [16] |
| Maximum risk of active TB mortality*(per month) | 0.0144 | Calibrated to provide DS-TB mortality |
| Probability of spontaneous resolution (per month) | 0.008 | [17] |
| Annual risk of relapse within 2 years of resolution (set to 0 afterward) | 0.02 (DS-TB)  0.04 (DR-TB) | [18–20] |
| Maximum probability of Smear conversion (negative to positive)* | 0.7 | [21] |
| TB care |  |  |
| Maximum probability of seeking care* (per month) | 0.176 | Calibrated to provide the DS-TB prevalence at baseline |
| Probability of DS-TB treatment in private sector | 0.5 | [11] |
| Probability of empiric treatment at initial encounter (among smear-negative patients) | 0.25 | Assumption |
| Probability of permanent loss-to-follow-up after initial encounter | 0.13 | [22] |
| Probability of returning for follow-up encounter (per month) | 0.46 | [22] |
| Probability of MDR-TB treatment failure | 0.17 | [23] |
| Sensitivity of Xpert for TB | 1.00 (smear-positive)  0.67 (smear-negative) | [24] |
| Sensitivity of Xpert for rifampin resistance | 0.95 |  |
| Specificity of Xpert | 0.99 |  |
| Transmission |  |  |
| Maximum DR-TB infectiousness (relative to DS-TB)* | 0.9 | Calibrated to provide DR-TB incidence at baseline |
| Individual’s risk of acquiring resistance after DS-TB treatment failure (post year 1976) | 6e-6 | Calibrated to provide target DR-TB incidence |
| Relative TB infectiousness for those failing treatment | 0.5 | Assumption |
| Infectiousness among children (<10 years old) | 0 | Assumption |
| Reduction in reinfection risk if latently infected | 0.5 | [25, 26] |
| Calibration targets | **Mean [lower-upper range]** |  |
| DS-TB incidence | 211 [109 – 345] per 100,000 people | [13] |
| DS-TB mortality | 32 [24 – 40] per 100,000 people |  |
| DR-TB incidence | 11 [7.2 – 15] per 100,000 people |  |
| Proportion of DR-TB in new patients | 0.028 [0.02 – 0.035] |  |
| Proportion of DR-TB in retreated patients | 0.12 [0.1 – 0.14] |  |

**Table S12: Summary of all simulation parameters and their values at baseline.**

* Parameter is modeled as a linear function of time since active disease development, reaching a maximum threshold at 9 months since infection and staying at that level afterward.

# Sensitivity analysis

## One-way sensitivity analyses

We performed univariate sensitivity analyses for selected epidemiological and cost-related parameters across the ranges shown in Table 1 in the main text. Figure S8 summarizes the results for main outcomes.


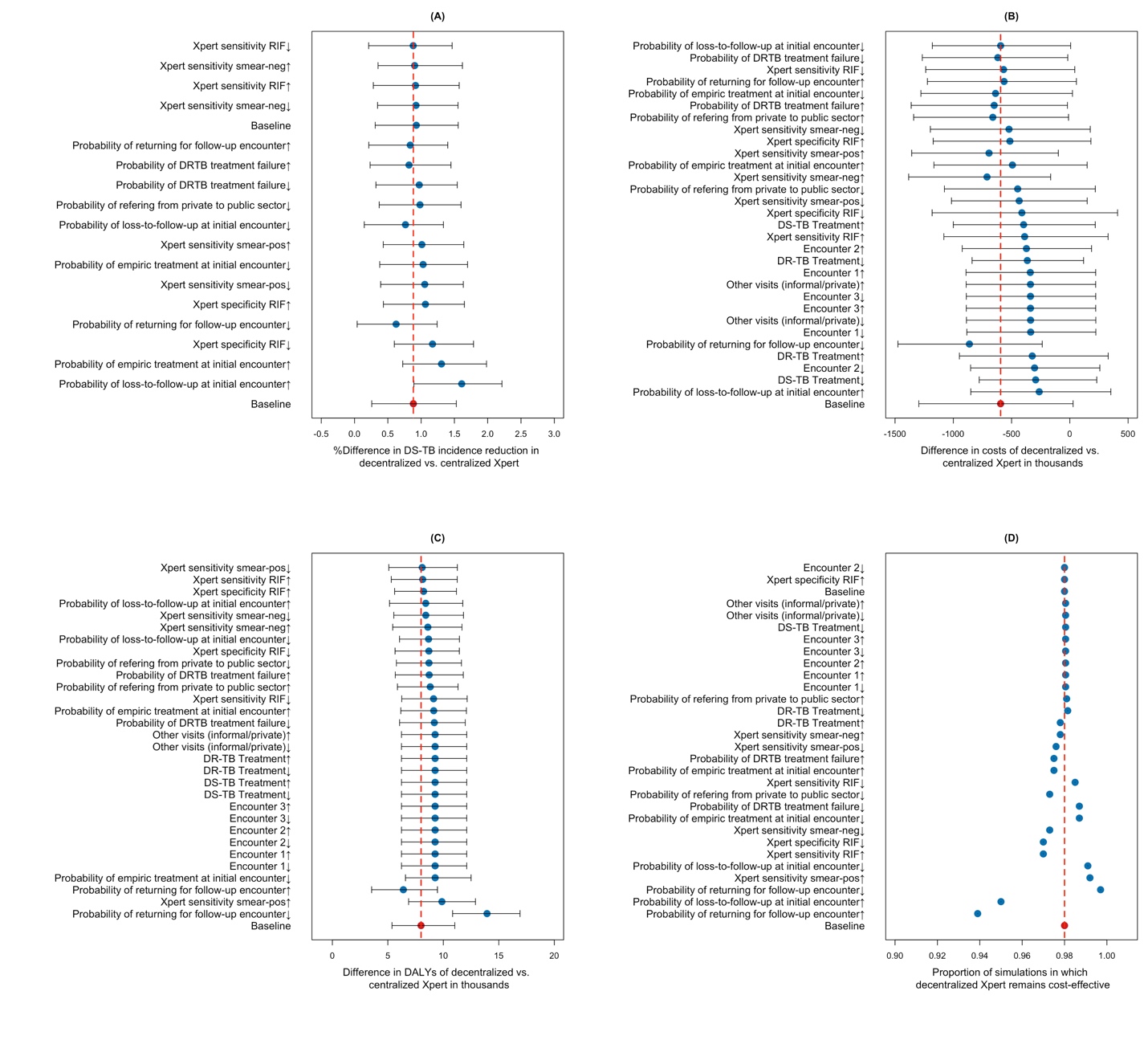
 **Figure S8. One-way sensitivity analysis to value of selected model parameters.** Panels show the sensitivity of epidemiological outputs (reduction in DS-TB incidence in panel A), costing outcomes (difference in costs in panel B, and DALYs averted in panel C) comparing decentralized to centralized TB testing, and the proportion of simulations that remain cost effective at a threshold of $1,000 (panel D) under one-way variation in value of selected model parameters. Each parameter value is followed by a up/down arrow, denoting an increase (↑) or decrease (↓) in the input parameter value as listed in Table 1. Each scenario is simulated starting in year 2018 and is followed to year 2028 under centralized and decentralized Xpert placement. The bars and arrows represent the median and interquartile ranges across 1000 simulations. The red mark and dashed line represent the baseline scenario with no parameter variation.

## Sensitivity and threshold analysis to incremental pre-treatment loss to follow up in the centralized scenario

Additional sensitivity analysis was performed to variation in probability of the pre-treatment loss-to-follow-up in the centralized scenario, assumed at 0.13 in main analysis and varied from 0% to 20% for sensitivity analysis. For costing purposes, we assumed a decentralized scenario with moderate volume of 3 tests per day and a centralized scenario with no cost sharing for sample transport (the most favorable scenario for decentralized Xpert) and $1000 threshold for willingness to pay. All results are summarized in Figure S9. Under higher levels of loss-to-follow-up in the centralized scenario, a smaller number of patients presenting to the public sector would successfully receive TB treatment, resulting in an increase in incidence of DS-TB and DR-TB when compared to a counterfactual scenario with no loss-to-follow-up (decentralized Xpert: Figure S9 Panel A&B). Increase in pre-treatment loss-to-follow-up was further associated with reduction in overall cost and DALYs (Panel C & D). This was further reflected in the final values of ICER, with higher proportion of simulations suggesting DXP as a cost-effective strategy at higher levels of pre-treatment loss-to-follow-up (Panel E).


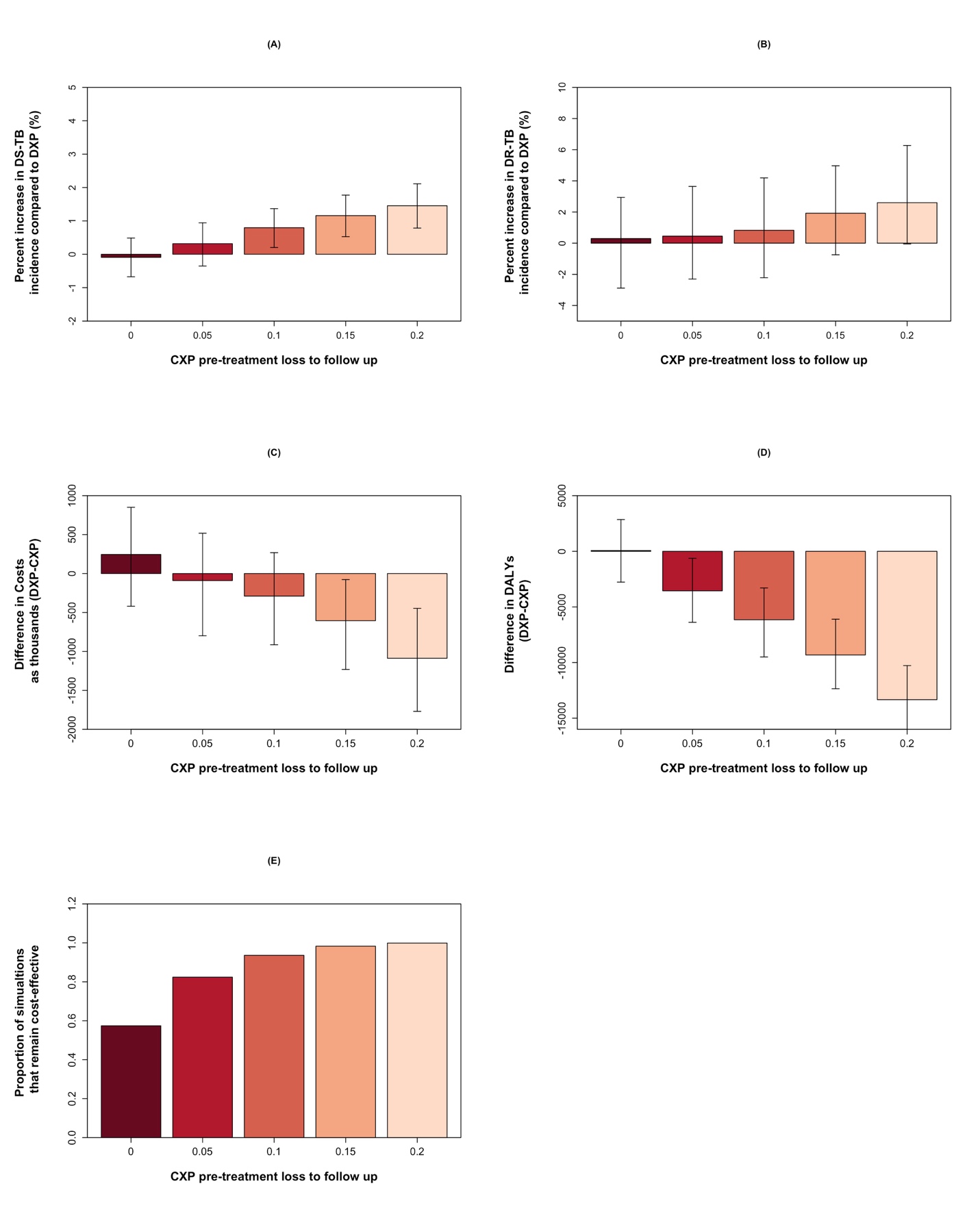


Figure S9: One-way sensitivity analysis of results to variation in probability of pre-treatment loss-to-follow-up in the centralized scenario compared to the decentralized scenario. The values in panel A to D represent medians and arrows mark the interquartile ranges of all simulations. Panel E represents the proportion of simulations in which decentralized testing remains cost-effective relative to centralized testing at a cost-effectiveness threshold of $1,000 per DALY averted. CXP, centralized Xpert testing; DXP, decentralized Xpert testing.

To further characterize the role of this parameter at different costing scenarios, we summarized the results in a threshold analysis format. Table S13 shows the threshold values (to the nearest 5%) for the incremental loss to follow-up in the centralized scenario at which 50% of simulations fall below the cost-effectiveness threshold, at various willingness to pay thresholds (rows) and various costing scenarios (columns). This table illustrates that, when no cost sharing of specimen transport network costs is possible and decentralized testing can be performed at a volume of 3 tests per day, decentralized testing is projected to be cost-effective in >50% of simulations, regardless of the cost-effectiveness threshold. By contrast, if decentralized testing can only be performed at a volume of 0.3 tests per day and the cost-effectiveness threshold is $1,000 per DALY averted or lower, decentralized testing is unlikely to be considered cost-effective under any scenario of incremental loss to follow-up. Cost-effectiveness assessments are most dependent on incremental loss to follow-up in the setting where decentralized testing can be performed at 3 tests per day, but specimen transport costs can be shared in the centralized testing scenario. In this case, at a cost-effectiveness threshold of $500 per DALY averted, decentralized testing was only deemed cost-effective (in 50% of simulations) if incremental loss to follow-up was 20% or higher in the centralized scenario. However, at a cost-effectiveness threshold of $2000 per DALY averted, decentralized testing would be deemed cost-effective if incremental loss to follow-up was only 5% or higher.

|  | Centralized specimen transport with 95%cost sharing | Centralized specimen  transport with no cost sharing | Centralized specimen transport with 95%cost sharing | Centralized specimen transport with no cost sharing | |
| --- | --- | --- | --- | --- | --- |
| Cost effectiveness threshold: | Decentralized test volume: 3 tests per day | | Decentralized test volume: 0.3 tests per day | | |
|  |  | |  | | |
| $500 per DALY averted | >20% | >0 | NA | NA | |
| $1,000 per DALY averted | >10% | >0 | NA | NA | |
| $2,000 per DALY averted | >5% | >0 | >20% | | >15% |

**Table S13**: Incremental loss to follow-up (centralized-decentralized) at which 50% simulations are below the cost effectiveness threshold, suggesting that decentralized testing is cost-effective relative to centralized testing. “>0” suggests that decentralized testing is considered cost-effective in 50% of simulations even if there is no difference in loss to follow-up between centralized and decentralized testing. “NA” suggests that, even if centralized testing incurs an additional 20% incremental loss to follow-up, decentralized testing will not be considered cost-effective at the corresponding cost-effectiveness threshold.

## Sensitivity analysis to role of smear

Additional sensitivity analysis was performed under the assumption that Xpert would be implemented in addition to smear (as opposed to the baseline scenario in which Xpert was implemented as a replacement for smear). In these simulations, all individuals presenting to the public sector for encounter 1 received a smear and Xpert test. In both centralized and decentralized scenarios, the Xpert results were prioritized over smear for TB diagnosis. However, we further assumed that individuals with smear-positive TB would not experience pre-treatment loss-to-follow-up in the centralized Xpert scenario. In this way, using smear as a back-up test in addition to Xpert will allow for immediate diagnosis and treatment of an additional fraction of patients who would otherwise go untreated in the centralized scenario. All results are summarized in Table S14 and Figure S10.

Using smear in addition to Xpert increased the epidemiological impact of centralized testing, as reflected by reduction in total DALYs from a median of 740,124 [IQR: 700,676 - 782,488] in baseline to 732,682 [IQR: 692,849 – 775,659] in the sensitivity analysis scenario. This resulted in a subsequent increase in the incremental cost-effectiveness ratio across all 4 costing scenarios (comparing ICER values in Table S14 to Table 2 in the main text), suggesting a lower preference for decentralized Xpert. Considering a willingness-to-pay threshold of $1,000 per DALY averted, the probability that decentralized testing would be cost-effective relative to centralized testing was 0.57 (compared to 0.98 in the primary analysis) assuming moderate decentralized testing volume and no cost-sharing for centralized specimen transport (Figure S10-solid blue line), and 0.07 (compared to 0.66) assuming moderate decentralized volume and 95% cost-sharing (Figure S10-solid red line).


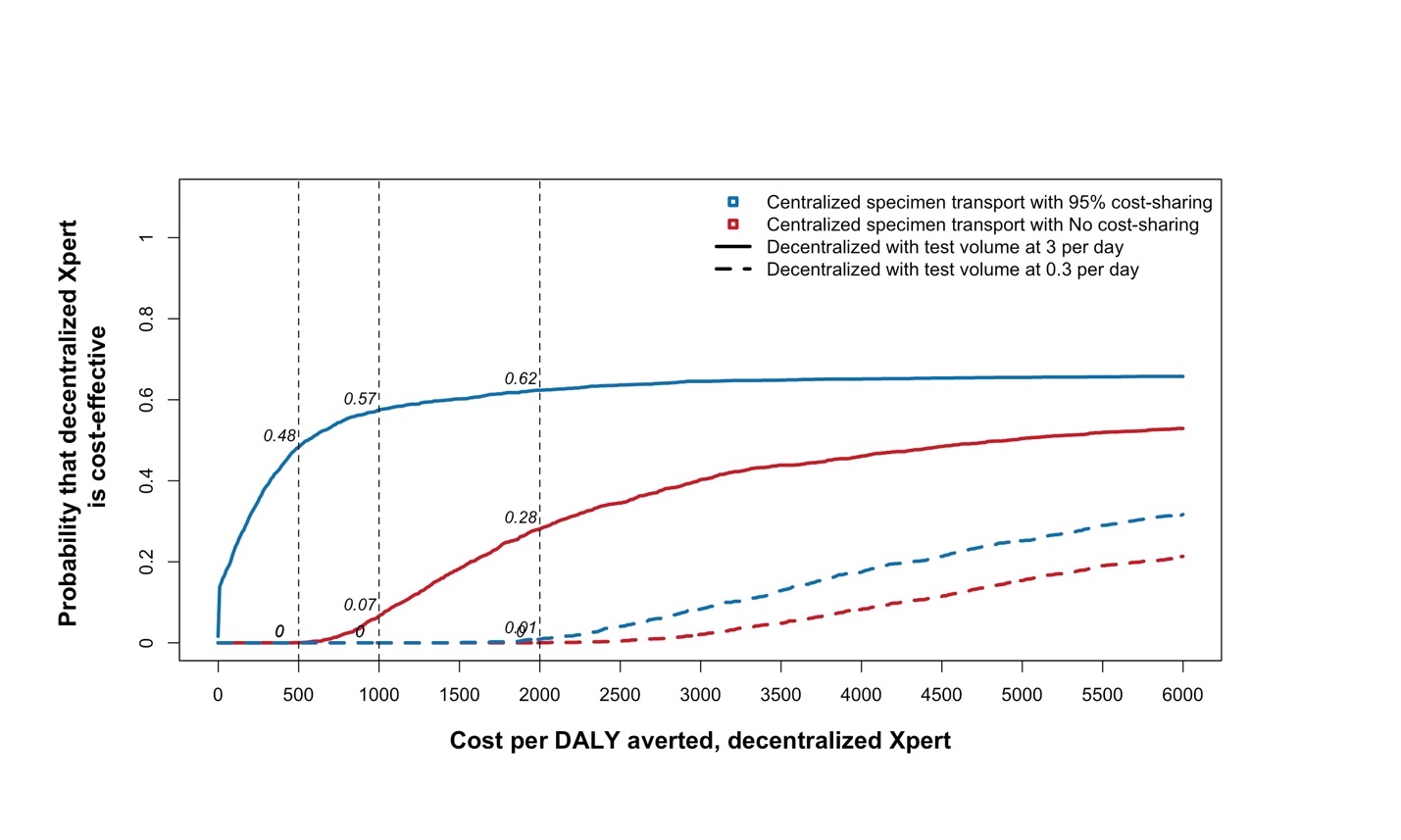


**Figure S10. Cost-Effectiveness acceptability curves comparing the centralized and decentralized Xpert cost scenarios when smear is used in addition to Xpert.** The x-axis shows the cost per DALY averted via decentralized Xpert compared to centralized testing and the y-axis shows the proportion of stochastic simulations falling below alternative cost-effectiveness thresholds (dotted black lines). Cost-effectiveness values are computed for the four unit-cost comparisons when centralized specimen transport is utilized at no vs. 95% cost sharing and testing volume ranges from 0.3 to 3 per day in the decentralized scenario.

|  | **Centralized Xpert with 95% cost-sharing for specimen transport** | | | **Centralized Xpert with no cost-sharing for specimen transport** | | **Decentralized Xpert with test volume at 3 per day** | | **Decentralized Xpert with test volume at 0.3 per day** | |
| --- | --- | --- | --- | --- | --- | --- | --- | --- | --- |
| **Total Costs**** | $187 | | | $195 | | $196 | | $217 | |
|  | [$156 – $221] | | | [$163 – $229] | | [$164 – $230] | | [$185 – $253] | |
| **Total DALYs** | 732,682 | | | | | 730,838 | | | |
|  | [692,849 – 775,659] | | | | | [692,027 – 773,548] | | | |
| **Comparing Centralized vs. Decentralized Xpert** | | | | | | | | | |
| **Centralized Xpert** | | **vs.** | **Decentralized Xpert** | | **Difference in costs**** | | **Difference in DALYs** | | **Cost per DALY averted** |
| 95% cost-sharing for specimen transport | | vs. | Test volume at 3 per day | | $8.0 | | 1,735  [-1,235 – 4,615] | | $4,915 |
|  |  |  |  |  | [$7.7 – $9.3] | |  |  | [1,812 – Inf*] |
| No cost-sharing for specimen transport | | vs. | Test volume at 3 per day | | $1.0 | |  |  | $589 |
|  |  |  |  |  | [$0.41 – $1.7] | |  |  | [135 – Inf*] |
| 95% cost-sharing for specimen transport | | vs. | Test volume at 0.3 per day | | $30 | |  |  | $17,348 |
|  |  |  |  |  | [$29 – $32] | |  |  | [6,629 – Inf*] |
| No cost-sharing for specimen transport | | vs. | Test volume at 0.3 per day | | $23 | |  |  | $13,033 |
|  |  |  |  |  | [$22 – $24] | |  |  | [4,946 – Inf*] |

**Table S14: Incremental Cost-Effectiveness of Decentralized Versus Centralized Xpert Testing when Xpert is implemented in addition to the smear.** A total of four cost sets were compared in the cost-effectiveness analysis. All values are expressed as median with inter-quartile ranges in the brackets, cumulative and discounted over a ten-year analysis frame.

* In these simulations, decentralized Xpert was less effective than centralized testing

** Cost estimates are expressed in units of million dollars

**References**

1. Sohn H, Puri L, Anh N, Nguyen T, Hoog AH Van, Nguyen VAT, Nliwasa M, Nabeta P (2019) Cost and affordability analysis of TB-LAMP and Xpert MTB / RIF assays as routine diagnostic tests in peripheral laboratories in Malawi and Vietnam. J Glob Heal Sci 1:1–11

2. Vassall A, van Kampen S, Sohn H, et al (2011) Rapid diagnosis of tuberculosis with the Xpert MTB/RIF assay in high burden countries: A cost-effectiveness analysis. PLoS Med 8:e1001120

3. Sarin S, Huddart S, Raizada N, et al (2019) Cost and operational impact of promoting upfront GeneXpert MTB/RIF test referrals for presumptive pediatric tuberculosis patients in India. PLoS One 14:e0214675

4. Cepheid | GeneXpert Omni. http://www.cepheid.com/us/genexpert-omni. Accessed 12 Feb 2018

5. Foundation for Innovative New Diagnsotics Negotiated Prices for Diagnostic Products in LMICs. https://www.finddx.org/find-negotiated-product-pricing/. Accessed 14 Nov 2018

6. Central TB Division (2015) TB India 2015, annual status report.

7. RNTCP (2017) NATIONAL STRATEGIC PLAN FOR TUBERCULOSIS ELIMINATION.

8. Sohn H, Vyas A, Puri L, Gupta S, Qin ZZ, Codlin A, Creswell J Costs and Operation Management of Community Outreach Program for Tuberculosis in Tribal Populations in India. Public Heal. Action (Submitted Rev.

9. Kasaie P, Sohn H, Kendall E, Gomez GB, Vassall A, Pai M, Dowdy DW (2017) Exploring the epidemiological impact of universal access to rapid tuberculosis diagnosis using agent-based simulation. In: 2017 Winter Simul. Conf. pp 1097–1108

10. Salje H, Andrews JR, Deo S, Satyanarayana S, Sun AY, Pai M, Dowdy DW (2014) The Importance of Implementation Strategy in Scaling Up Xpert MTB/RIF for Diagnosis of Tuberculosis in the Indian Health-Care System: A Transmission Model. PLoS Med 11:e1001674

11. Kapoor SK, Raman AV, Sachdeva KS, Satyanarayana S (2012) How did the TB patients reach DOTS services in Delhi? a study of patient treatment seeking behavior. PLoS One 7:1–6

12. Mistry N, Rangan S, Dholakia Y, Lobo E, Shah S (2016) Durations and Delays in Care Seeking , Diagnosis and Treatment Initiation in Uncomplicated Pulmonary Tuberculosis Patients in Mumbai , India. 1–17

13. World Health Organization (2018) Global Tuberculosis Report 2018.

14. E. VYNNYCKY, M. FINE PE (1997) The natural history of tuberculosis: the implications of age-dependent risks of disease and the role of reinfection. Epidemiol Infect 119:183–201

15. Marais BJ, Gie RP, Schaaf HS, Hesseling AC, Obihara CC, Nelson LJ, Enarson DA (2004) The clinical epidemiology of childhood pulmonary tuberculosis : a critical review of literature from the pre-chemotherapy era. 8:278–285

16. Horsburgh Jr. CR (2004) Priorities for the treatment of latent tuberculosis infection in the United States.[see comment]. N Engl J Med 350:2060–2067

17. Tiemersma EW, van der Werf MJ, Borgdorff MW, Williams BG, Nagelkerke NJD (2011) Natural history of tuberculosis: Duration and fatality of untreated pulmonary tuberculosis in HIV negative patients: A systematic review. PLoS One. doi: 10.1371/journal.pone.0017601

18. Marx FM, Dunbar R, Enarson D a, Williams BG, Warren RM, van der Spuy GD, van Helden PD, Beyers N (2014) The temporal dynamics of relapse and reinfection tuberculosis after successful treatment: a retrospective cohort study. Clin Infect Dis 58:1676–83

19. Lienhardt C, Paydar A, Menzies D, Benedetti A, Martin I, Pai M, Burman W, Vernon A, Royce S (2009) Effect of Duration and Intermittency of Rifampin on Tuberculosis Treatment Outcomes: A Systematic Review and Meta-Analysis. PLoS Med 6:e1000146

20. Palmero DJ, Ambroggi M, Brea A, et al (2004) Treatment and follow-up of HIV-negative multidrug-resistant tuberculosis patients in an infectious diseases reference hospital, Buenos Aires, Argentina. Int J Tuberc Lung Dis 8:778–784

21. Organization WH (2016) Global Tuberculosis Report 2016.

22. Macpherson P, Houben MGJ, Glynn JR, Corbett L, Kranzer K (2014) Systematic Systematic reviews reviews Pre-treatment loss to follow-up in tuberculosis patients in low- and lower-middle-income countries and high-burden countries : a systematic review and meta-analysis. Bull World Health Organ 126–138

23. Organization WH (2017) Global Health Observatory Data Repository. http://apps.who.int/gho/data/?theme=main&vid=60740.

24. Steingart KR, Schiller I, Horne DJ, Pai M, Boehme CC, Dendukuri N (2014) Xpert ® MTB / RIF assay for pulmonary tuberculosis and rifampicin resistance in adults ( Review ) Xpert ® MTB / RIF assay for pulmonary tuberculosis and rifampicin resistance in adults. Cochrane Libr. doi: 10.1002/14651858.CD009593.pub3.Copyright

25. Andrews JR, Noubary F, Walensky RP, Cerda R, Losina E, Horsburgh CR (2012) Risk of progression to active tuberculosis following reinfection with Mycobacterium tuberculosis. Clin Infect Dis 54:784–791

26. Sutherland I, Švandová E, Radhakrishna S (1982) The development of clinical tuberculosis following infection with tubercle bacilli. Tubercle 63:255–268

1. Costs estimated based on figures provided by Dr. Dean Sher at SmartSpotQ in South Africa, a company that provides EQA to public and private TB laboratories using Xpert tests in South African and other countries (including India). For GX4 and Omni, costs were assessed based on three EQA panel testing for a set of 4 individual GeneXpert modules (1 GX4 = 4 modules; 1 Omni = 1 module). [↑](#footnote-ref-1)
2. Percent indicates exclusivity of sample transport network for TB. Lower value represents high cost sharing of sample transport with other clinical samples whereas 100% indicates exclusive TB sample transport network for TB. [↑](#footnote-ref-2)
